# SET/PP2A signaling regulates macrophage positioning in hypoxic tumor regions by amplifying chemotactic responses

**DOI:** 10.1038/s12276-022-00867-0

**Published:** 2022-10-12

**Authors:** Shaolong Zhang, Jingping Zhou, Pengzhao Shang, Guomeng Zhao, Anlei Wang, Jinlei Mao, Yuhang Tao, Ziyi Chen, Xuehao Wang, Changying Guo

**Affiliations:** 1 School of Life Science and Technology, China Pharmaceutical University, Nanjing, PR China; 2 Hepatobiliary/Liver Transplantation Center, The First Affiliated Hospital of Nanjing Medical University, Nanjing, PR China

**Keywords:** Immunoediting, Monocytes and macrophages

## Abstract

Tumor-associated macrophages (TAMs) are one of the main cellular components in the tumor microenvironment (TME). In many types of solid tumors, TAMs tend to accumulate in hypoxic areas and are intimately related to poor patient prognosis. However, the underlying mechanisms by which TAMs infiltrate hypoxic tumor regions remain unclear. In this study, we report that genetic deletion of SE translocation (SET) in myeloid cells inhibited the entry of TAMs into the hypoxic tumor region and abated their proangiogenic and immunosuppressive functions, ultimately inhibiting tumor growth. Mechanistically, in response to hypoxic tumor supernatant stimulation, SET in macrophages shuttled between the nucleus and cytoplasm via the PKC-CK2α signaling axis. Cytoplasmic retention of SET increased ERK and P38 signaling by inhibiting PP2A, which promoted TAM migration into the hypoxic area and polarization toward the M2 phenotype. Therefore, we conclude that SET modulates tumor immunity by acting as a key regulator of macrophage positioning and function in the tumor.

## Cancer: reducing recruitment of tumor-promoting cells

Deleting the multifunctional protein SET inhibits tumor growth by suppressing migration to tumors of tumor-associated macrophages (TAMs), immune cells that have been turned against the body to promote tumor growth and block antitumor immune cells. Tumors alter their environment to boost their own growth, decreasing oxygen levels and recruiting cells such as TAMs. Changing Guo at China Pharmaceutical University Nanjing, Ziyi Chen and Xuehao Wang at The First Affiliated Hospital of Nanjing Medical University, China, and co-workers investigated how TAMs are recruited. Deletion of SET decreased TAM levels in hypoxic tumor regions and increased antitumor immunity in a mouse model. They conclude that deletion of SET prevents TAMs from detecting signals from the tumor microenvironment, thereby decreasing TAM recruitment and suppressing tumor growth. SET is a promising target for cancer immunotherapy.

## Introduction

In addition to tumor cells, solid tumors also contain a variety of immune cells and stromal cells, which form a complex tumor microenvironment (TME)^1^. Inside solid tumors, malignant cell proliferation, altered cell metabolism, and disordered tumor blood vessels reduce the transport of oxygen and nutrients, resulting in tumor hypoxia^2^. Accumulating evidence suggests a link between hypoxia and tumor tolerance to immune surveillance through the recruitment of a large number of immunosuppressive cells, such as tumor-associated macrophages (TAMs), myeloid-derived suppressor cells (MDSCs), and regulatory T cells (Tregs)^3,4^. Furthermore, multiple clinical studies have shown that hypoxia correlates with the poor prognosis of cancer patients^5^.

As the most abundant immune cells, TAMs account for more than 50% of infiltrated leukocytes in some tumors^6^. Plasticity is a hallmark of macrophages that enables the cells to exhibit different phenotypes in response to environmental cues, exerting pro- or antitumor activity. Increasing evidence shows that TAMs mainly accumulate in avascular, necrotic/hypoxic areas in tumors^7^. To date, various mechanisms of TAM recruitment and retention in the hypoxic TME have been demonstrated. For example, hypoxia increases the levels of chemoattractants, which activate corresponding receptors, triggering the phosphorylation of downstream signaling pathways, including P38 and extracellular-signal-regulated kinase (ERK), and promoting TAM recruitment to hypoxic regions^7^. Casazza et al. reported that TAM entry into hypoxic tumor areas is regulated by semaphorin 3 A/neuropilin-1 signaling. Hypoxia upregulates semaphorin 3 A and vascular endothelial growth factor (VEGF), which act as macrophage attractants by inducing neuropilin-1-dependent or neuropilin-1-independent VEGFR1 transactivation to drive TAM localization into hypoxic areas. Once macrophages reach the hypoxic zone, chemokine receptors, such as CCR2, CCR5, and neuropilin-1, significantly decrease, terminating the macrophage migratory response^8^. Furthermore, hypoxia-mediated upregulation of MAPK phosphatase 1 (MPK1) inhibits chemokine-activated ERK and P38 signaling, resulting in macrophages remaining in the hypoxic zone^9^. When positioned in hypoxic tumor regions, TAMs alter their gene expression profiles and signaling pathways, leading to the development of a distinct protumor phenotype. For example, TAMs upregulate growth factors, such as FGF2, PDGF, and VEGF, to promote angiogenesis and tumor growth in nutrient-deficient regions^10^. In addition, TAMs secrete a series of matrix metalloproteinases (MMPs), such as MMP2, MMP7, and MMP9, which promote tumor migration and invasion^11^. TAMs in hypoxic regions also enhance immunosuppression by inhibiting T-cell activation. HIF1α directly induces programmed cell death 1 ligand 1 (PD-L1) expression on TAMs, which subsequently suppresses T-cell effector function by binding to its receptor (PD-1) on T cells^12^. Additionally, TAMs recruit CCR4-expressing Tregs to the tumor by secreting cytokines such as CCL17 and CCL22^13^. Therefore, reprogramming TAMs by changing their intratumoral distribution is thought to be a sufficient and feasible approach for cancer immunotherapy.

SE translocation (SET), which is also known as inhibitor 2 of protein phosphatase 2A (I2PP2A), is a multifunctional oncoprotein that participates in many cellular processes, including DNA replication, DNA repair, the cell cycle, and transcription. SET is highly expressed in a variety of tumors and promotes the occurrence, development, and metastasis of tumors^14^. Clinical data show that the expression level of SET is negatively correlated with the overall survival of cancer patients^15^. SET was initially defined as a nuclear protein, but increasing evidence shows that SET is also distributed in the plasma membrane and cytoplasm. The subcellular location of SET is intimately associated with its biofunctions. For example, nuclear SET functions as a histone chaperone that is required for assembling/disassembling nucleosomes during transcription. SET also inhibits the activity of histone acetyltransferases by binding and masking histones. In the cytoplasm, SET interacts with a series of cytoplasmic proteins, including PP2A, NM23-H1, and Rho GTPase Rac family small GTPase 1 (Rac1), promoting cell spreading and migration^14^.

According to the Human Protein Atlas program, SET is also extensively and highly expressed in a variety of immune cells. However, the fact that SET is overexpressed in myeloid leukemias, including chronic myeloid leukemia (CML) and acute myeloid leukemia (AML), prompted us to examine the physiological function of SET in myeloid cells, particularly in TAMs. Herein, we report that genetic deletion of SET significantly impairs macrophage entry into hypoxic tumor regions and potentiates antitumor immunity in a syngeneic mouse tumor model. We demonstrate that in response to a hypoxic environmental cue, SET translocates to the cytoplasm through the protein kinase C (PKC)-casein kinase 2α (CK2α) signaling axis and regulates macrophage migration by promoting the activation of ERK and P38 by inhibiting PP2A. Our findings establish SET as a key regulator of macrophage positioning and function within tumors and suggest that SET is a promising target for cancer immunotherapy.

## Materials and methods

### Mouse and cell culture

B6.I2PP2A^flox/flox^ (SET^*fl/fl*^) mice were generated by Bangyao Biotechnology Co., Ltd (Shanghai, China). B6.LysM-Cre^+/−^ mice were obtained from Fudan University Medical College, Shanghai. The LLC and B16F10 cell lines were purchased from Zeye Biology Co., Ltd. RAW264.7 cells were obtained from ATCC. Cells were maintained in complete DMEM (10% fetal bovine serum (Homeland, China), 200 mM l-glutamine, and 100 units/ml penicillin‒streptomycin). All cells used in this study were cultured at 37 °C with 5% CO_2_.

### Syngeneic tumor model

For the tumor model, LLC cells (5 × 10^5^) or B16F10 cells (2 × 10^5^) were subcutaneously injected into the shaved flanks of recipient mice. Tumor dimensions were measured using a caliper starting on Day 7 and every 2 days thereafter. Tumor volume was calculated by using the formula (ab^2^) π/2, where a is the longest measurement and b is the shortest. Tumor tissues were harvested after 2 weeks.

### Real-time RT‒PCR and RNA-seq

Total RNA was isolated from cells by TRIzol reagent, and first-strand cDNA was synthesized using a HiScript 1st Strand cDNA Synthesis Kit (Vazyme, China). Real-time qPCR was performed in triplicate using SYBR Green Mix (Vazyme, China) on an Applied Biosystems 7500 Real-time System. The primer sequences are listed in Supplementary Table 1. For RNA-seq, BMDMs derived from WT and L/L mice were prepared, and total RNA was extracted using TRIzol reagent. RNA library preparation and RNA sequencing were performed by Novogene (Tianjin, China).

### Flow cytometric analysis and cell sorting

Tumors were harvested and digested using a cocktail consisting of collagenase (Worthington, USA) and DNase I (Sigma-Aldrich, USA). Single-cell suspensions were then filtered through a 70-μm cell strainer. After the erythrocytes were lysed twice, the cells were resuspended in PBS. Then, the samples were incubated for 5 min at room temperature with an anti-mouse CD16/32 Fc Block. The single-cell suspension was stained with BD Horizon™ Fixable Viability Stain 700 (FVS700) (BD Biosciences, USA) for dead cell discrimination. After being washed, the surface markers of the cells were stained with the indicated antibodies (CD45, CD3, CD4, CD8, CD11b, LY6G, F4/80, CD206, and CD163) for 30 min on ice in the dark. After being stained, the cells were washed again with PBS and resuspended in PBS for flow cytometric analysis. The results were analyzed by BD FACS Diva Software (BD Biosciences, USA).

For TAM sorting, the tumor single-cell suspension was stained with CD45, CD11b, LY6G, and F4/80 for 30 min on ice. The population of CD45^+^CD11b^+^LY6G^-^F4/80^+^ (tumor-associated macrophages) was sorted by a BD FACSAria II SORP flow cytometer (BD Biosciences, USA). The antibodies used in this study are listed in Supplementary Table 2.

### Intracellular cytokine staining

Tumor single-cell suspensions were stained with BD Horizon™ Fixable Viability Stain 700 (FVS700), and surface markers were stained. Then, the cells were permeabilized and fixed with BD Cytofix/Cytoperm™ Fixation/Permeabilization Solution (BD Biosciences, USA) at 4 °C for 20 min. The cells were washed twice with 1× Perm/Wash buffer and stained with fluorescence-conjugated intracellular cytokine antibodies, such as anti-IFNγ-APC, at 4 °C for 30 min. The cells were washed again and resuspended in PBS for flow cytometric analysis.

### T-cell proliferation assay

Mouse splenic T cells were isolated from a nontumor-bearing C57BL/6 mouse using BD IMag™ Mouse T Lymphocyte Enrichment Set-DM (BD Biosciences, USA). T cells were labeled with CFSE (5 µM) and nonspecifically activated with anti-CD3/CD28 beads (Life Technologies). TAMs (sorted from WT and L/L mouse tumors) were cocultured with T cells at a 1:5 ratio. After 3 days, the cells were harvested, and the CFSE signal in the gated CD8^+^ T cells was measured by flow cytometry.

### Isolation of neutrophils from mouse spleen

Then, 65 and 78% of Percoll solutions were prepared. A single-cell suspension of WT mouse spleen cells was prepared, followed by erythrocyte lysis. The single-cell suspension was slowly added to the prepared Percoll solution and centrifuged at 500×*g* for 30 min. A pipette was used to withdraw the layer of white cells between the two densities, which contained neutrophils.

### Western blotting, immunohistochemistry, and immunofluorescence staining

Western blotting was performed according to the standard protocol. The primary antibodies and dilutions used were as follows: SET (1:1000), phospho-ERK1/2 (1:1000), ERK1/2 (1:1000), P38 (1:1000), p-P38 (1:1000), and GAPDH (1:3000). Antibody information is listed in Supplementary Table 2.

Tumors were harvested, extensively washed with PBS, and immediately immersed in 4% paraformaldehyde. The fixed tissue was sent to Wuhan Servicebio Biotechnology Co., Ltd. (Wuhan, China), for immunohistochemistry and immunofluorescence staining. Polychromatic immunofluorescence analysis was performed with F4/80, CD31, CAIX, and DAPI to show the intratumoral location of TAMs. Polychromatic immunofluorescence analysis was performed with F4/80, CD8, CAIX, and DAPI to show the intratumoral location of CD8^+^ T cells.

### Induction of bone marrow-derived macrophages (BMDMs)

Bone marrow was isolated from the long bones of euthanized mice. After the erythrocytes were lysed, the single-cell suspension was prepared by filtration through a 70-μm cell strainer. Bone marrow cells were then cultured in a 1640 medium containing M-CSF (20 ng/ml). After 3 days, the medium was replaced with a fresh 1640 medium containing 10% FBS serum and M-CSF (20 ng/ml). The cells were cultured for another 4 days. The adherent cells were bone marrow-derived macrophages (BMDMs). For BMDM polarization, the M1 phenotype was induced with of IFNγ (20 ng/ml, Peprotech, USA), whereas the M2 phenotype was induced with IL4 (40 ng/ml, Peprotech, USA), as previously described.

### Transwell migration assay

Transwell assays were performed in 24-well plates with inserts (5-μm pore size; Millipore, USA). Briefly, 5 × 10^4^ BMDMs or peritoneal macrophages were cultured in the upper chamber, while the lower chamber contained 600 μl of hypoxic tumor supernatant. After 2 h of incubation at 37 °C, cells that migrated through the polycarbonate membrane were fixed with methanol and stained with crystal violet. The cells were counted in five random fields of view.

### Hoechst 33342 staining

Hoechst 33342 staining was used as a vascular marker to detect acute hypoxia in tumors. Briefly, to detect tumor hypoxia, 50 mg/kg Hoechst 33342 (Sigma-Aldrich, USA) was administered intravenously for 20 min before the mice were sacrificed. Then, a tumor single-cell suspension was prepared as described previously, and macrophages were analyzed by flow cytometry.

### Intratumoral distribution of BMDMs

Bone marrow from WT and L/L littermates was isolated and induced in vitro for 7 days to obtain BMDMs. BMDMs from WT mice were stained with DiD dye (10 μM) for 1 h. BMDMs from L/L mice were stained with CFSE dye (10 μM) for 1 h. BMDMs from WT and L/L mice were collected after being washed three times with DMEM. BMDMs from WT and L/L mice were counted and mixed at a 1:1 ratio. Finally, the cell mixture (4 × 10^6^) was intravenously injected into WT mice that had been subcutaneously transplanted with LLC cells for 2 weeks. Two days after the injection, a Hoechst 33342 staining assay was performed to examine the intratumoral distribution of BMDMs.

### Statistical analysis

Statistical comparisons of the datasets were performed by two-tailed Student’s *t*-test, one-way ANOVA with Tukey’s posttest, or two-way ANOVA with Bonferroni’s correction using Prism software (Version 4.00; GraphPad Inc.). Data were considered statistically significant when *p* < 0.05.

## Results

### The loss of SET in myeloid cells significantly inhibits tumor growth

**Fig. 1:**
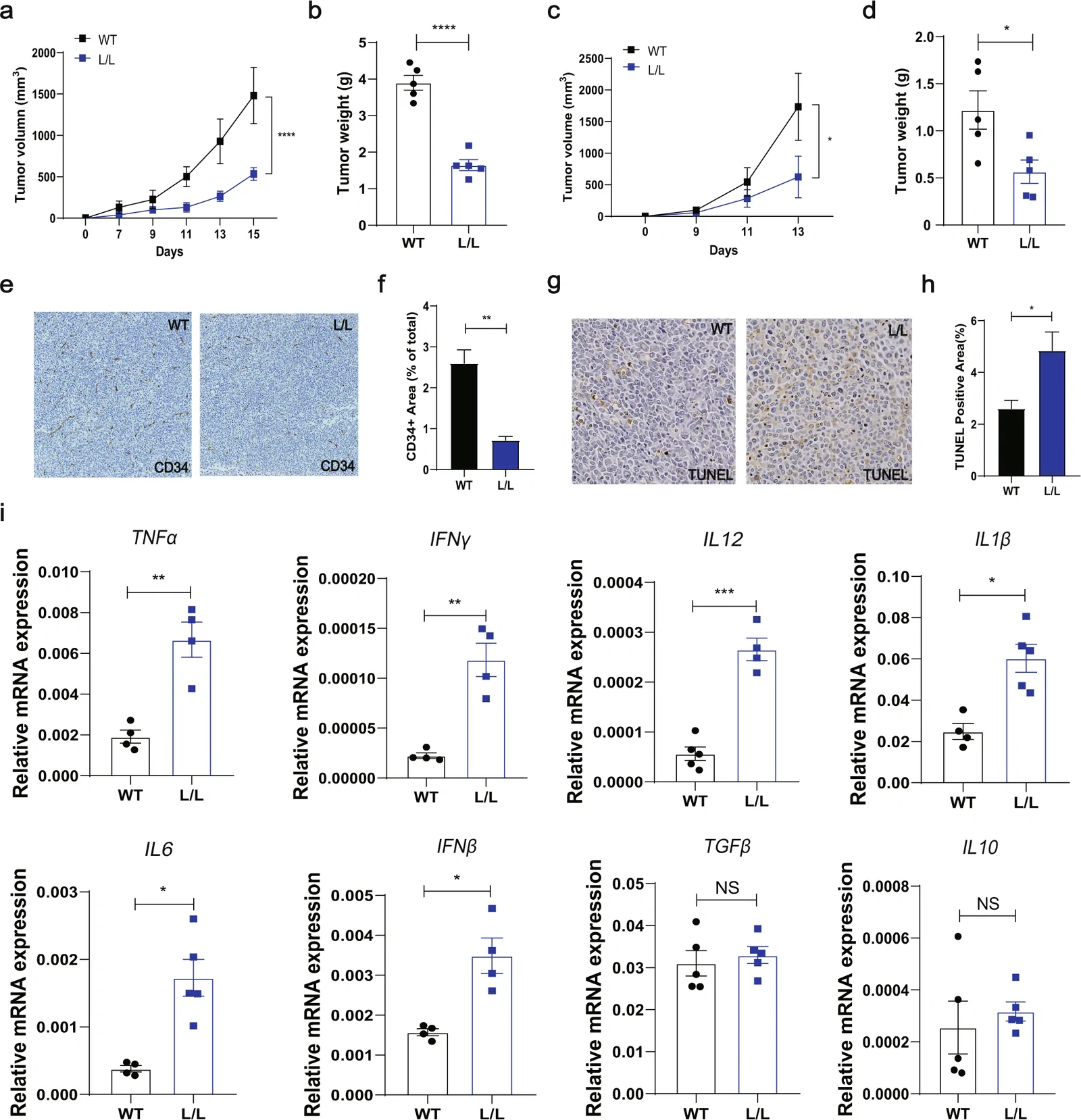
The loss of SET in myeloid cells reduces tumor burden. **a**, **b** Subcutaneous LLC tumor volume (**a**) and weight (**b**) in mice with myeloid cell-specific deletion of SET (LysM-Cre^+/−^, SET^*fl/fl*^; L/L) and control mice (LysM-Cre^−/−^, SET^*fl/fl*^; WT) (*n* = 5). one-way ANOVA with Tukey’s posttest. *p < 0.05, **p < 0.01, ***p < 0.001, ****p < 0.0001. **c**, **d** Subcutaneous B16F10 tumor volume (**c**) and weight (**d**) in mice with myeloid cell-specific deletion of SET (L/L) and control mice (WT) (*n* = 5). **e**, **f** Immunohistochemical staining of CD34 in tumor blood vessels in LLC tumor tissue sections from WT and L/L mice. Representative images (**e**) and statistical analysis of CD34 (**f**) are shown. **g**, **h** TUNEL assays showing apoptosis in LLC tumor tissue sections from WT and L/L mice. Representative images (**g**) and statistical analysis of the quantified signals (**h**) are shown. Scale bar, 20 μm. **i** The mRNA levels of selected cytokines, such as *TNFα*, *IFNγ*, *IL12*, *IL1β*, *IL6*, *IFNβ*, *TGFβ,* and *IL10*, in LLC tumor tissues from WT and L/L mice were examined by RT‒PCR. The RT‒PCR data were the mean ± SEM of at least three biological replicates. Student’s *t*-test. *p < 0.05, **p < 0.01, ***p < 0.001, ****p < 0.0001.

To investigate the role of myeloid SET in tumor immunity, we generated a mouse model with inducible myeloid SET ablation by crossbreeding LysM-Cre and SET ^fl/fl^ mice (Supplementary Fig. 1a). LysM-Cre^+/−^ SET^fl/fl^ (L/L) mice and LysM-Cre^−/−^ SET^fl/fl^ (WT) mice were used in subsequent experiments (Supplementary Fig. 1b). Routine blood tests showed that the loss of SET in myeloid cells did not affect the proportion of neutrophils, lymphocytes, monocytes, eosinophils, or basophils in the blood (Supplementary Fig. 1c). There were no significant abnormalities in other routine blood indices (data not shown), indicating that SET knockout in myeloid cells had little effect on the physiological state of the mice. To examine whether SET in myeloid cells is involved in tumor immunity, Lewis lung carcinoma (LLC) cells were subcutaneously injected into littermate WT and L/L mice. As shown Fig. 1a, b, the loss of SET in myeloid cells significantly reduced tumor volume and weight. A similar antitumor effect was also observed in the B16F10 syngeneic mouse model (Fig. 1c, d). Angiogenesis and apoptosis in LLC tumor tissues were examined by CD34 immunohistochemistry and TUNEL assays. Compared to those in WT mice, reduced angiogenesis (Fig. 1e, f) and increased tumor cell apoptosis were observed in L/L mice (Fig. 1g, h). Next, we evaluated the state of the immune microenvironment in L/L mice. Total RNA was extracted from tumor tissue, and the expression levels of a series of cytokines were determined. We found that LLC tumors in L/L mice showed increased levels of typical antitumor cytokines, such as *TNFα*, *IFNγ*, *IL12*, *IL1β*, *IL6*, and *IFNβ*, while the immunosuppressive cytokines *IL10* and *TGFβ* remained unchanged (Fig. 1i). We also evaluated the immune microenvironment in the B16F10 tumor model. Similarly, B16F10 tumors in L/L mice exhibited increased expression of proinflammatory cytokines such as *TNFα*, *IFNγ*, *IL12*, *IL1β*, and *IL6* and reduced expression of anti-inflammatory cytokines such as *IL10*, *IL4*, and *TGFβ* (Supplementary Fig. 2). These results suggest that SET deletion in myeloid cells significantly potentiates antitumor immunity.

### The loss of SET in TAMs promotes immune surveillance

**Fig. 2:**
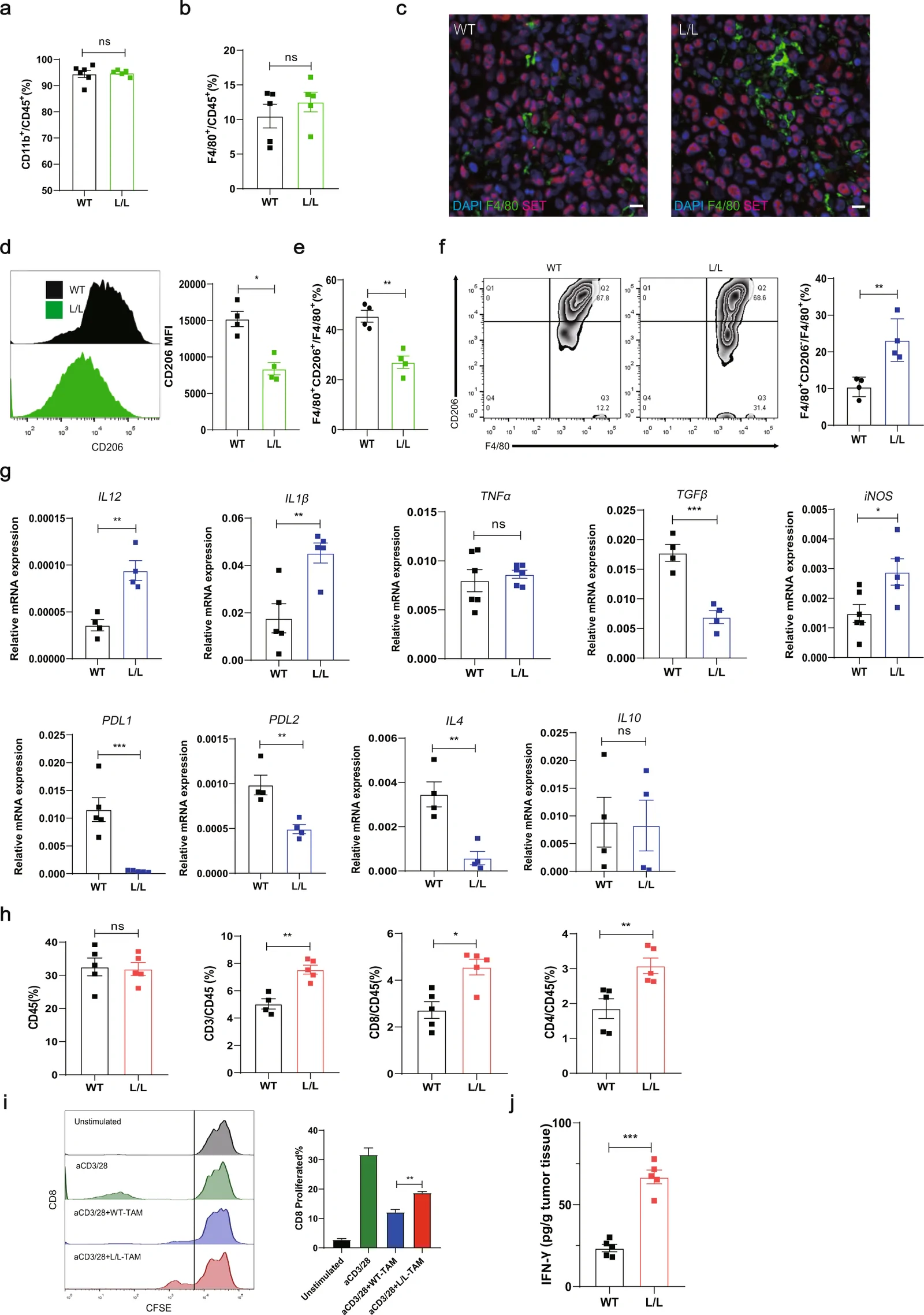
The loss of SET in TAMs promotes immune surveillance. **a**, **b** the Loss of SET in myeloid cells has no effect on LLC tumor-infiltrating monocytes (**a**) or TAMs (**b**), as determined by FACS analysis. **c** Immunofluorescence staining showing SET knockout in TAMs. Scale bar, 20 μm. **d** Expression levels of CD206 in TAMs in LLC tumor tissues from WT and L/L mice, as determined by flow cytometry. **e**, **f** Flow cytometric analysis of the proportion of CD206^+^ cells in TAMs in LLC tumor tissues from WT and L/L mice. (**e** cell surface CD206 staining; **f** intracellular CD206 staining). **g** The mRNA level of M1 macrophage gene expression (*INOS*, *IL12*, *IL1β*, and *TNFα*) and M2 macrophage gene expression (*TGFβ*, *PD-L1*, *PD-L2*, *IL4*, and *IL10*) in TAMs sorted from the subcutaneous LLC tumors of WT mice and L/L mice. The data are the mean ± SEM of at least three biological replicates. Student’s *t*-test. *p < 0.05, **p < 0.01, ***p < 0.001, ****p < 0.0001. **h** FACS analysis of LLC tumor-infiltrating T-cell proportions (CD45^+^, CD3^+^, CD4^+^, and CD8^+^ cells) from WT and L/L mice. **i** The frequency of proliferated CD8^+^ T cells was examined in vitro in coculture experiments with TAMs. TAMs were sorted from the LLC tumors or WT and L/L mice (*n* = 5 tumors). The data shown here are representative of three independent experiments. **j** The concentration of IFNγ in tumor homogenate from WT and L/L mice was measured by ELISA. The data were the mean ± SEM of at least three biological replicates. Student’s *t-*test. *p < 0.05, **p < 0.01, ***p < 0.001, ****p < 0.0001.

We then investigated the composition of the immune infiltrate in LLC tumors from WT and L/L mice. First, the effect of SET ablation on myeloid cells in tumors was examined. The gating strategies for the analysis of myeloid cells and TAMs in tumor tissue are shown in Supplementary Fig. 3a. We found that there were no differences in the proportions of myeloid cells and TAMs between WT and L/L mice, suggesting that myeloid-specific SET deletion did not affect monocyte recruitment or macrophage differentiation from monocytes (Fig. 2a, b). We further confirmed this effect using bone marrow-derived macrophages (BMDMs) induced by macrophage colony-stimulating factor (M-CSF) in vitro. The knockout efficiencies of SET in BMDMs and peritoneal macrophages were validated by RT‒PCR and western blotting, respectively (Supplementary Fig. 3b, c). Neither total macrophages (F4/80^+^ cells) nor mature macrophages (F4/80^+^LY6C^−^ cells) changed in the absence of SET (Supplementary Fig. 3d, e). Correspondingly, SET depletion did not affect the mRNA levels of *PU.1*, *IRF8*, or *Csf1R* in BMDMs, which are closely related to macrophage maturation (Supplementary Fig. 3f). Taken together, our data suggest that SET is not functionally related to either the recruitment of monocytes or the differentiation of monocytes into macrophages within the tumor.

Next, we focused on the impact of SET knockout on the TAM phenotype within the tumor. SET knockout in TAMs was validated by immunofluorescence analysis (Fig. 2c). The expression level of CD206 on the TAM surface was markedly decreased, and the proportion of CD206^+^ TAMs (intracellular staining of CD206) was also attenuated (Fig. 2d–f). The gene expression profile of TAMs that were sorted by flow cytometry showed that loss of SET significantly upregulated the expression of genes such as *INOS*, *IL12*, and *IL1β* and downregulated *TGFβ*, *IL4, PD-L1*, and *PD-L2* expression (Fig. 2g). These results suggest that SET deletion promotes the polarization of TAMs toward the M1 phenotype.

The immune status of TAMs profoundly influences T-cell function^16^. It is particularly noteworthy that the expression of PD-L1 in the TAMs of L/L mice was downregulated more than tenfold compared to that in WT mouse TAMs. Thus, we analyzed the infiltration and activation of T cells. The gating strategy for the analysis of T cells in tumor tissue is shown in Supplementary Fig. 4a. The loss of SET in myeloid cells significantly promoted the infiltration of CD3^+^, CD4^+^ and CD8^+^ T cells in LLC tumors (Fig. 2h). In the B16F10 tumor model, the proportion of CD3^+^ cells was unchanged, while CD8^+^ and activated CD8^+^ T cells, which express IFNγ (CD8^+^IFNγ^+^), were significantly increased in L/L mice (Supplementary Fig. 4b–d). To further demonstrate the relationship between TAMs and T-cell activation, a coculture assay was performed. The sorted TAMs from WT or L/L mice were coincubated with T cells at a ratio of 1:5 for 3 days. We found that the TAMs of L/L mice significantly promoted the proliferation of CD8^+^ T cells compared with those from WT mice (Fig. 2i). IFNγ not only promotes the M1 polarization of macrophages but is also a marker of CD8^+^ T-cell activation. IFNγ levels in tumor tissue extract from L/L mice were significantly higher than those in WT mice (Fig. 2j). These results demonstrate that the loss of SET in myeloid cells promotes the immune activation of TAMs and increases the infiltration and activation of CD8^+^ T cells.

### The loss of SET in neutrophils barely affects their plasticity in the TME

In the LysM-Cre system, the Cre enzyme is expressed in the entire myeloid cell population, indicating that SET is also depleted in neutrophils. We found that neutrophils also accounted for a significant proportion of the immune infiltrate in the TME, but SET knockout did not affect the proportion of neutrophils (Supplementary Fig. 5a). We isolated neutrophils from the spleens of WT and L/L mice and examined SET expression at both the mRNA and protein levels. The mRNA level of SET in neutrophils was significantly lower than that in BMDMs and peritoneal macrophages (Supplementary Fig. 5b, c). Correspondingly, the SET protein level in neutrophils was significantly lower than that in BMDMs and peritoneal macrophages (Supplementary Fig. 5c). It has been reported that neutrophils in the TME undergo polarization similar to TAMs and can be divided into N1 and N2 phenotypes, which exert antitumor and protumor effects, respectively^17^. The phenotypes of neutrophils in the TME were analyzed by flow cytometry; LY6G^+^LY6C^High^ labeled N1 cells and LY6G^+^LY6C^Low^ labeled N2 cells^18^. The results showed that the percentage of N1 neutrophils was not different between L/L mice and WT mice (Supplementary Fig. 5d). These results demonstrate that SET expression in neutrophils is significantly lower than that in macrophages, and the loss of SET in neutrophils has little effect on their phenotype in the TME.

### Genome-wide analysis of SET-mediated transcriptional regulation in BMDMs

**Fig. 3:**
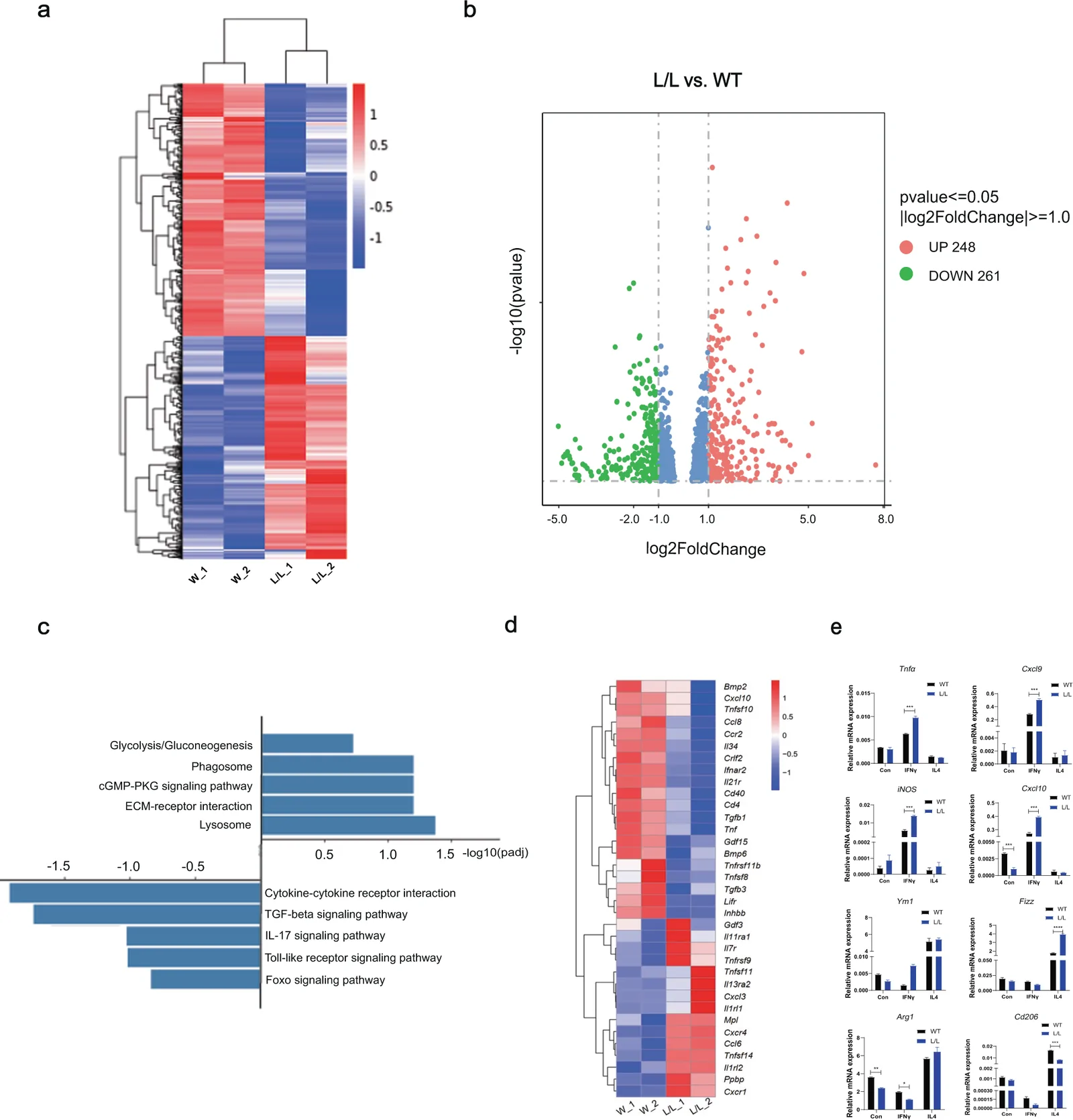
Genome-wide analysis of SET-mediated transcriptional regulation in BMDMs. Heatmap (**a**) and a volcano plot (**b**) showing the differentially expressed genes (DEGs) in BMDMs derived from WT mice and L/L mice. **c** KEGG pathway analyses of all DEGs in BMDMs from WT mice and L/L mice. **d** Heatmap showing a subset of upregulated or downregulated genes involved in cytokine‒cytokine receptor interactions. **e** The mRNA levels of the selected gene sets in BMDMs derived from WT and L/L mice stimulated with IFNγ (20 ng/ml) or IL4 (20 ng/ml) for 24 h. The data were the mean ± SEM of at least three biological replicates. Student’s *t*-test. *p < 0.05, **p < 0.01, ***p < 0.001, ****p < 0.0001.

To comprehensively understand the function of SET in macrophages, we examined differential gene expression in BMDMs derived from WT and L/L mice using RNA-seq. Hierarchical clustering was used to organize the genes by expression patterns across samples after RNA-seq analysis (Fig. 3a). The volcano plot showed that the loss of SET upregulated 248 genes and downregulated 261 genes (Fig. 3b). KEGG pathway enrichment analysis showed that SET deletion significantly inhibited cytokine‒cytokine receptor interactions, the TGFβ signaling pathway, and the IL17 signaling pathway (Fig. 3c). We focused on the differentially expressed genes (DEGs) involved in the cytokine‒cytokine receptor interaction pathway, as shown by the heatmap (Fig. 3d). CC and CXC chemokines trigger the movement of monocytes, natural killer cells, dendritic cells, and T cells^19^. SET has complicated effects on the expression of CC chemokine ligands (CCLs). SET deletion significantly downregulated *CXCL10* and *CCL8* expression and upregulated *CXCL3* and *CCL6* expression (Fig. 3d). We also found that these DEGs exhibited different expression patterns between BMDMs and TAMs in vivo. However, SET deletion downregulated TGFβ expression in both BMDMs and TAMs (Figs. 2g, 3d), which plays a critical role in promoting M2 macrophage activation^20^.

To investigate whether SET affects cytokine-induced macrophage polarization, we examined the expression of different gene signatures in M1 and M2 BMDMs after the cells were induced with various cytokines. The polarization of BMDMs toward the M1 or M2 phenotype is usually induced by IFNγ or IL4, respectively^21^. Compared to WT BMDMs, BMDMs with SET deletion expressed higher levels of *iNOS*, *TNFα*, *CXCL10*, and *CXCL9* in response to IFNγ stimulation, indicating that SET deletion amplified the IFNγ signal in macrophages in vitro (Fig. 3e). In response to IL4 induction, the expression levels of genes associated with the M2 phenotype, including *Ym1*, *Arg1*, *Fizz,* and *CD206*, robustly increased regardless of whether SET was present. However, SET deletion exerted complicated effects on the upregulation of IL4-induced genes. For example, SET deletion augmented IL4-induced *Fizz* upregulation but had no significant effect on *Ym1* or *Arg1* expression (Fig. 3e). These results indicate that SET differentially regulates the expression of M1- and M2-related genes. Collectively, our data suggest that SET-mediated transcriptional regulation in macrophages does not affect macrophage plasticity.

### The loss of SET impairs the spatial distribution of TAMs within tumors

**Fig. 4:**
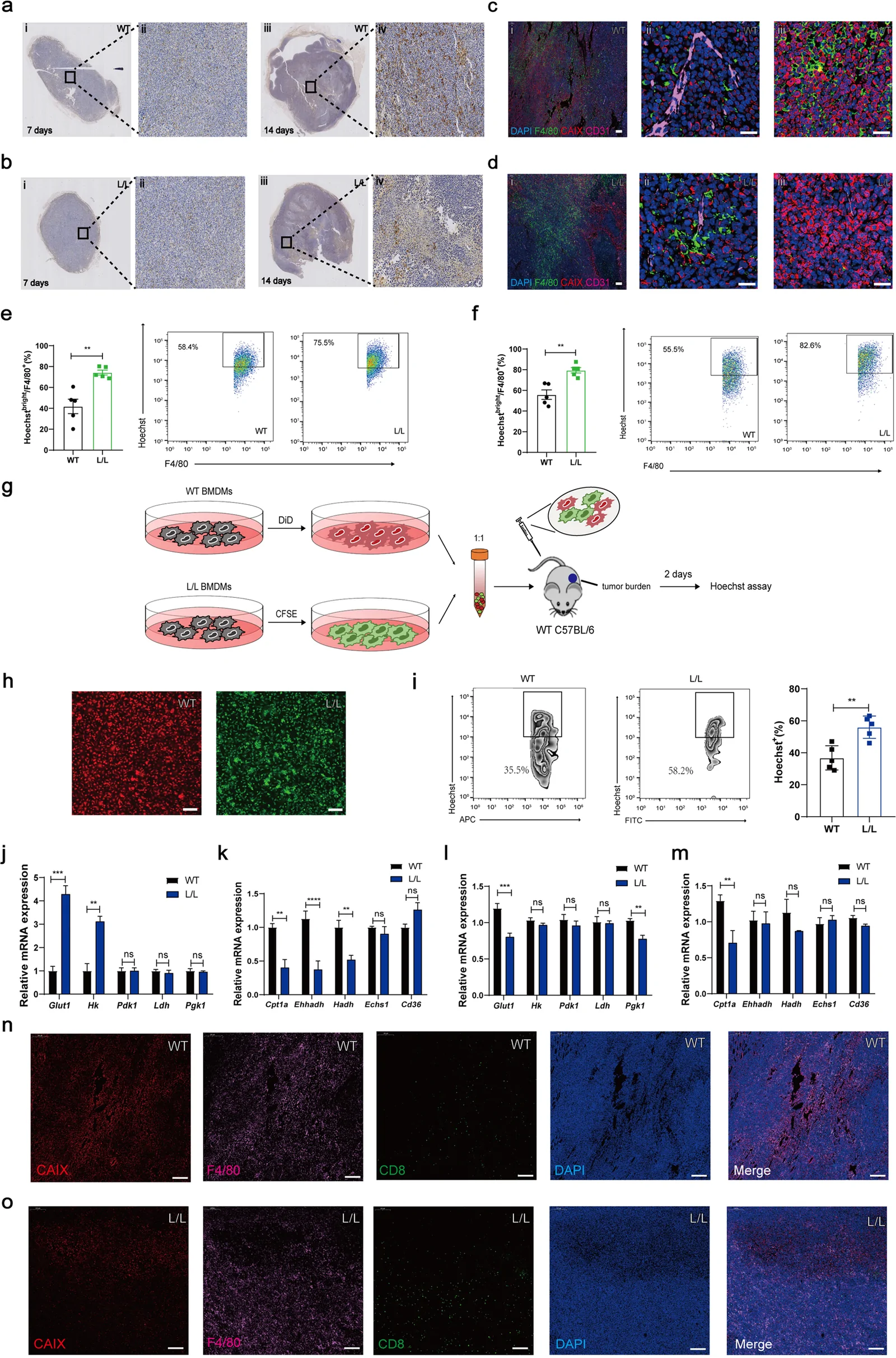
The loss of SET impairs the spatial distribution of TAMs within tumors. **a**, **b** IHC analysis showing LLC tumor-infiltrating TAMs from WT (**a**) and L/L mice (**b**) in the early and end-stage. Early-stage (i), magnified partial view (ii represents the internal area of i); End-stage (iii), magnified partial view(iv represents the necrotic area of iii). Scale bar, 50 μm. **c**, **d** Immunofluorescence staining of CAIX (red), F4/80 (green), and CD31 (pink) in tumor tissue sections from WT (**c**) and L/L mice (**d**). The merged images are shown in (i). Scale bar, 200 μm. The right two panels are magnified local views of the normoxic region (ii) and hypoxic region (iii). Scale bar, 20 μm. **e**, **f** FACS analysis of aerobic and hypoxic TAM proportions in LLC tumors (**e**) and B16F10 tumors (**f**) from WT and L/L mice. Hoechst^bright^ F4/80^+^ cells are classified as aerobic TAMs, and Hoechst^dim^ F4/80^+^ cells are classified as hypoxic TAMs. At least five biological replicates were performed. Student’s *t*-test. *p < 0.05, **p < 0.01. **g**, **h** Schematic diagram showing the intratumoral distribution of BMDMs with the indicated staining in vivo. Representative micrographs showing the staining of BMDMs from WT (red) mice and L/L (green) mice. Scale bar, 20 μm. **i** FACS analysis showing the proportions of aerobic and hypoxic BMDM in LLC tumors from WT and L/L mice. Hoechst^bright^DiD^+^ cells were classified as aerobic BMDMs from WT mice, and Hoechst^bright^CFSE^+^ cells were classified as aerobic BMDMs from L/L mice. **j**, **k** The mRNA levels of glycolysis-related genes (**j**), such as *Glut1*, *HK*, *PDK1*, *LDH*, and *PGK1*, and fatty acid oxidation-related genes (**k**), such as *CPT1A*, *EHHDH*, *HADH*, *ECHS1*, and *CD36*, in TAMs sorted from subcutaneous LLC tumors from WT and L/L mice. **l**, **m** The mRNA levels of glycolysis-related genes (**l**) and fatty acid oxidation-related genes (**m**) in BMDMs derived from WT and L/L mice. **k**–**m** All data show the mean ± SEM of at least three biological replicates. Student’s *t*-test. *p < 0.05, **p < 0.01, ***p < 0.001, ****p < 0.0001. **n**, **o** Immunofluorescence staining of CAIX (red), F4/80 (pink), and CD8 (green) in tumor tissue sections from WT (**n**) and L/L mice (**o**). Scale bar, 50 μm.

In the literature, the positioning of TAMs within tumors determines their antitumor or protumor activity^22^. We sought to examine the effect of spatial distribution on macrophage phenotype and function. We analyzed the distribution of macrophages at different stages of tumor growth. After establishing the LLC allograft model, tumor tissues were harvested on the 7th and 14th days. Immunohistochemical analysis with F4/80 antibodies showed that there was no difference in macrophage distribution in the early stage of tumor progression between WT and L/L mice. However, during the late stage, the tumors implanted in L/L mice exhibited more apoptotic and necrotic regions where the population of macrophages was significantly reduced (Fig. 4a, b). Necrosis is one of the characteristics of hypoxic areas^23^. As the tumor grows, a large number of hypoxic areas appear inside the tumor. Therefore, we hypothesized that TAMs in L/L mice may have a different distribution in the hypoxic region compared with WT mice. CAIX, which is a target gene of HIF1A, is often used as a marker of hypoxic regions. Multicolor immunofluorescence staining of CAIX (hypoxic zone), CD31 (blood vessel), and F4/80 (macrophages) was performed to analyze the distribution of TAMs in hypoxic regions. We found that most TAMs in the tumor tissues of WT mice were located in hypoxic areas stained by CAIX (Fig. 4c). Surprisingly, TAMs in the tumor tissues of L/L mice were distributed away from hypoxic areas and were located around blood vessels (Fig. 4d). Hoechst 33342, an avascular perfusion dye, has been used to differentiate hypoxic cells within tumor tissue by FACS according to brightness^24^. After intravenous administration of Hoechst for a period of time, cells away from blood vessels were Hoechst^dim^, while cells in proximity to blood vessels were Hoechst^bright^. Consistent with the immunofluorescence staining results, flow cytometric analysis validated that the Hoechst^bright^F4/80^+^ proportion of TAMs in L/L mice was significantly higher than that in WT mice (Fig. 4e). The same results were observed in the B16F10 tumor model (Fig. 4f). Taken together, our data suggest that the loss of SET significantly reduces the distribution of TAMs in hypoxic regions.

To exclude the influence of individual animal differences on macrophage chemotactic ability in the TME, we assessed the positioning of BMDMs derived from WT and L/L mice simultaneously within the tumor. BMDMs from WT mice were stained red with DiD dye, and BMDMs from L/L mice were stained green with CFSE (Fig. 4g, h). The two populations of BMDMs were mixed in a 1:1 ratio and then injected into the tail veins of tumor-bearing WT mice. The Hoechst 33342 assay was performed 2 days after injection. Both kinds of BMDMs were allowed to distribute into the same tumor microenvironment. The proportion of BMDMs from WT or L/L mice was analyzed by flow cytometry. We then examined the Hoechst-positive ratio of the two types of BMDMs. We found that the number of Hoechst-positive BMDMs from L/L mice was significantly higher than that of BMDMs from WT mice (Fig. 4i). These results further suggested that the loss of SET could significantly impair the chemotaxis of macrophages toward hypoxic regions.

The metabolic state of macrophages is also influenced by the surrounding environment. Reportedly, fatty acid oxidation is increased in macrophages in hypoxic regions, while macrophages in normoxic regions prefer glycolysis for their energy supply^25^. A series of genes related to glycolysis and fatty acid oxidative phosphorylation (FAO) were analyzed in sorted TAMs from the tumor. We found that the expression of the glycolysis-related genes *Glut1* and *HK* was increased in L/L mice (Fig. 4j), while the expression of the FAO-related genes *CPT1A*, *EHHDH*, and *HADH* was decreased (Fig. 4k). To exclude the effect of SET knockout on the transcription of these genes, we examined changes in the mRNA levels of these metabolic genes in BMDMs. SET knockout did not affect the mRNA levels of these genes in BMDMs, which was further confirmed by our transcriptome data (Fig. 4l, m). Changes in the expression of genes involved in glycolysis and FAO further suggest that SET deletion affects the spatial positioning of TAMs.

It has been reported that the recruitment of T cells in tumors is associated with macrophages^26^. Given that SET deletion affects the spatial location of macrophages within tumors, we examined whether the distribution of T cells would change correspondingly. We performed polychromatic immunofluorescence staining of CD8^+^ T cells, macrophages, and hypoxic regions. As shown in Fig. 4n, o, the distribution of CD8^+^ T cells in the hypoxic region was significantly decreased in L/L mice, and most of these cells were colocalized in the normoxic region with TAMs. This result suggests that the changes in TAM location caused by SET deletion may disturb the position of other immune cells.

### SET regulates the chemotaxis of macrophages through P38 and ERK signaling

**Fig. 5:**
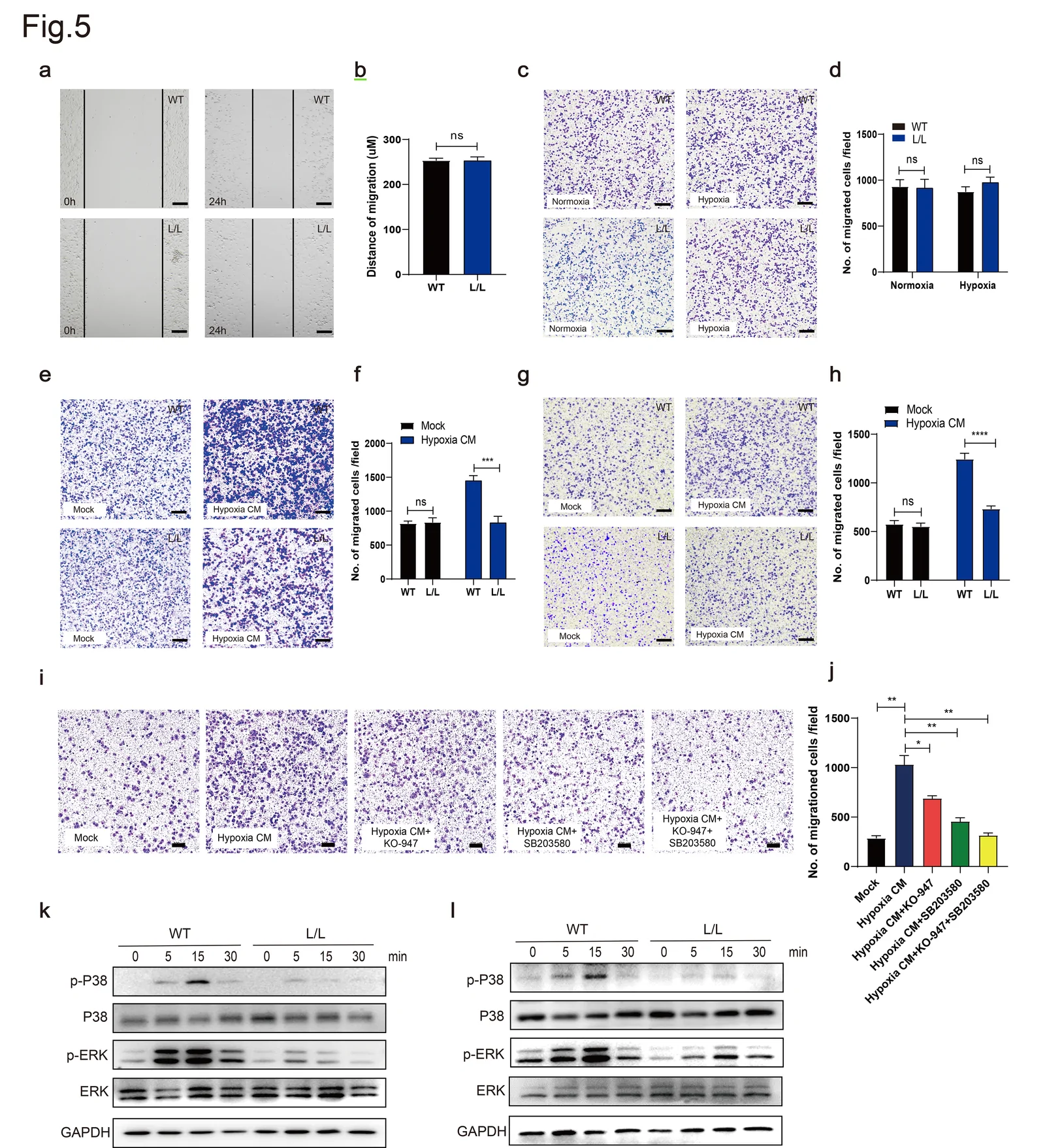
SET regulates chemotaxis in macrophages through p38 and ERK signaling. **a**, **b** Representative images (**a**) and quantitative analysis (**b**) of wound healing tests of cultured BMDMs derived from WT L/L mice. Scale bar, 100 μm. Student’s *t*-test. **c**, **d** Representative images (**c**) and quantitative analysis (**d**) of transwell migration assays of BMDMs derived from WT and L/L mic mice under normoxic and hypoxic conditions. The cells were allowed to migrate for 2 h at 37 °C before being stained with crystal violet. Scale bar, 100 μm. Student’s *t*-test. **e**, **f** Representative images (**e**) and quantitative analysis (**f**) of transwell migration assays of BMDMs derived from WT and L/L mice; culture medium was used as the mock condition, and hypoxic LLC tumor supernatant was used as hypoxic CM. Scale bar, 100 μm. Student’s *t*-test. **g**, **h** Representative images (**g**) and quantitative analysis (**h**) of transwell migration assays of peritoneal macrophages from WT and L/L mice; culture medium was used as the mock condition, and hypoxic LLC tumor supernatant was used as hypoxic CM,. Scale bar, 100 μm. Student’s *t*-test. **i**, **j** Representative images (**i**) and quantitative analysis (**j**) of transwell migration assays of BMDMs derived from WT and L/L mice; culture medium was used as the mock condition, and hypoxic LLC tumor supernatant was used as hypoxic CM. Scale bar, 100 μm. ERK inhibitor KO-947 (10 µM) and P38 inhibitor SB203580 (10 µM) were added in the experiment. Student’s *t*-test. **k**, **l** Western blot showing the effect of hypoxic LLC tumor supernatant on the activation of ERK and P38 in BMDMs (**k**) or peritoneal macrophages (**l**) derived from WT and L/L mice.

Next, we examined the regulatory effects of SET on the distribution of TAMs in the hypoxic region. First, we investigated the motility of macrophages in the absence of SET using a scratch experiment. SET knockout had no significant effect on the motility of macrophages (Fig. 5a, b). Second, we examined the motility of macrophages under hypoxic and normoxic conditions by transwell migration assays. The loss of SET did not affect the motility of macrophages under normal or hypoxic conditions, suggesting that the impaired entry of macrophages in the hypoxic region caused by a SET deletion in vivo is not attributed to changes in their motility (Fig. 5c, d). Thus, we hypothesized that SET deletion mitigated the macrophage response to chemotactic signals, which suppressed the entry of macrophages into the hypoxic region. To address our hypothesis, we investigated the chemotaxis of BMDMs in response to hypoxic LLC supernatant. LLC cells were cultured under hypoxic conditions to prepare hypoxic tumor supernatant. The loss of SET significantly inhibited BMDM chemotaxis toward hypoxic LLC supernatant (Fig. 5e, f). The same results were obtained in peritoneal macrophages (Fig. 5g, h).

It has been reported that a variety of chemokines secreted by hypoxic tumor regions can bind corresponding receptors on the surface of macrophages and promote the migration of macrophages by activating downstream ERK and P38 signaling pathways^27^. First, we verified this idea by examining the motility of BMDMs in the presence of the ERK inhibitor KO-947 (10 µM) or the P38 inhibitor SB203580 (10 µM). The ERK and P38 inhibitors inhibited the chemotaxis of macrophages toward the hypoxic tumor supernatant. The combination of ERK and P38 inhibitors synergistically inhibited macrophage chemotaxis toward hypoxic tumor supernatant (Fig. 5i, j). Therefore, we hypothesized that SET knockout may affect the activation of ERK and P38 signaling in macrophages and regulate macrophage chemotaxis toward hypoxic tumor supernatants. Consistent with the previous literature^28^, we found that hypoxic tumor supernatant rapidly and significantly activated ERK and P38 signaling in BMDMs from WT mice. However, in SET-deficient BMDMs, ERK and P38 signaling was weakly activated (Fig. 5k). The same results were obtained in peritoneal macrophages (Fig. 5l). Additionally, SET deletion inhibited the activation of ERK and P38 induced by hypoxic B16F10 supernatant (Supplementary Fig. 6a). Taken together, our data suggest that SET deletion in macrophages impairs the activation of ERK and P38 signaling induced by hypoxic tumor supernatant and thus blocks the chemotaxis of macrophages toward hypoxic supernatant.

### Macrophage chemotaxis is regulated by SET and relies on PP2A inhibition

**Fig. 6:**
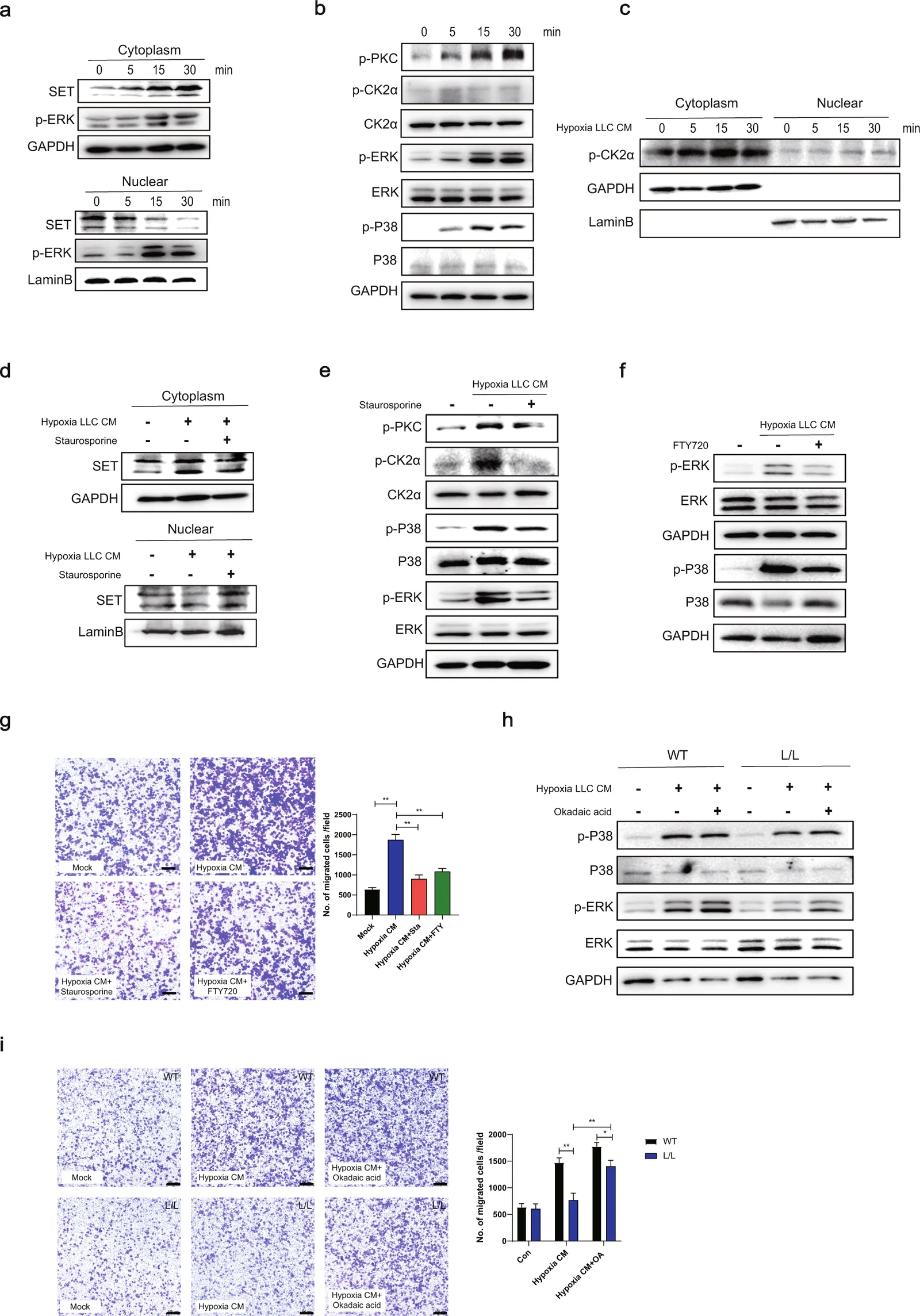
Macrophage chemotaxis is regulated by SET and relies on PP2A inhibition. **a** Subcellular distribution of SET in RAW264.7 cells in response to hypoxic LLC tumor supernatant stimulation at the indicated time point was detected by western blotting. **b** The activation of PKC, CK2α, ERK, and P38 signaling in RAW264.7 cells in response to hypoxic LLC tumor supernatant stimulation at the indicated time points was detected by western blotting. **c** The activation of CK2α in the nucleus and cytoplasm in RAW264.7 cells in response to hypoxic LLC tumor supernatant stimulation at the indicated time points was detected by western blotting. **d** The effect of staurosporine (100 nM) (the selective inhibitor of PKC) on SET redistribution in RAW264.7 cells in response to hypoxic LLC tumor supernatant stimulation was detected by western blotting. **e** The effect of staurosporine (100 nM) on the activation of PKC, CK2α, ERK, and P38 in RAW264.7 cells in response to hypoxic LLC tumor supernatant stimulation was detected by western blotting. **f** The effect of FTY720 (5 µM) on the activation of ERK and P38 in RAW264.7 cells in response to hypoxic LLC tumor supernatant stimulation was detected by western blotting. **g** Representative images (left panel) and quantitative analysis (right panel) of transwell migration assays showing the effects of staurosporine (100 nM) and FTY720 (5 µM) on the migration of BMDMs toward hypoxic LLC tumor supernatant. The cells were allowed to migrate for 2 h at 37 °C before being stained with crystal violet. Scale bar, 100 μm. Student’s *t*-test. **h** Okadaic acid (100 nM) promoted the activation of ERK and P38 in BMDMs derived from WT and L/L mice, as determined by western blotting. **i** Representative images (left panel) and quantitative analysis (right panel) of transwell migration assays showing the effects of okadaic acid (100 nM) on the migration of BMDMs from WT and L/L mice toward hypoxic LLC tumor supernatant. Scale bar, 100 μm. Student’s *t*-test.

PP2A, which is a serine/threonine phosphatase, is extensively involved in various cellular processes, including protein synthesis, signal transduction, cell cycle determination, apoptosis, metabolism, and stress response^29–32^. Moreover, PP2A can directly inhibit cervical cancer cell migration by dephosphorylating p-JNK, p-P38, and the p-ERK/MAPK^33^. Given that SET is a natural inhibitor of PP2A, we further investigated whether SET regulates macrophage chemotaxis through PP2A inhibition. First, we examined the subcellular location of SET in macrophages in response to hypoxic tumor supernatant. RAW264.7 cells were stimulated with hypoxic LLC tumor supernatant, and nuclear and cytoplasmic fractions were prepared. Western blotting showed that hypoxic tumor supernatant significantly promoted the translocation of SET from the nucleus to the cytoplasm (Fig. 6a). Reportedly, PKC activates CK2α and participates in the regulation of ERK and P38 signaling^34–37^. CK2α is mainly responsible for SET phosphorylation at serine 9, which leads to cytoplasmic retention and nuclear export of SET^38^. Thus, we examined the phosphorylation levels of PKC and CK2α in RAW264.7 cells treated with hypoxic tumor supernatant. ERK and P38 were significantly activated during treatment. As expected, the phosphorylation levels of PKC and CK2α increased gradually in the presence of the hypoxic tumor supernatant (Fig. 6b). Moreover, the activation of CK2α in the nucleus and cytoplasm was detected. The phosphorylation of CK2 was increased in both the cytoplasm and nucleus by hypoxic tumor supernatant (Fig. 6c). Staurosporine, a selective PKC inhibitor, was used to assess the role of PKC in SET translocalization. Staurosporine almost completely blocked the nuclear export of SET induced by hypoxic tumor supernatant (Fig. 6d). In addition, staurosporine significantly inhibited the activation of CK2α, ERK, and P38 (Fig. 6e). Taken together, these results suggest that hypoxic tumor supernatant activates PKC by phosphorylation and promotes the nuclear export of SET, which subsequently regulates ERK and P38 activation.

Theoretically, cytoplasmic SET can inhibit PP2A, which attenuates the phosphorylation of ERK and P38. We further investigated whether the inhibition of ERK and P38 signaling by a SET deletion in macrophages was PP2A dependent. FTY720, a sphingosine analog drug, has been approved by the FDA as a treatment for multiple sclerosis (MS)^39^. FTY720 reactivates PP2A by interacting with SET and preventing the SET–PP2Ac association^40^. Pretreatment of BMDMs with FTY720 significantly inhibited the activation of ERK and P38 induced by hypoxic tumor supernatant (Fig. 6f). Next, we examined the effect of staurosporine and FTY720 on macrophage migration toward hypoxic tumor supernatant. As shown in Fig. 6g, staurosporine and FTY720 both significantly reduced the migration of macrophages toward the hypoxic tumor supernatant. The microbial toxin okadaic acid (OA) specifically inhibits PPP-type serine/threonine protein phosphatases^41^. Thus, we investigated whether OA could counteract the inhibition of the ERK and P38 signaling caused by SET deletion. We found that OA treatment significantly enhanced the activation of ERK and P38 signaling in BMDMs derived from L/L mice stimulated with hypoxic tumor supernatant (Fig. 6h). In the transwell migration assay, OA significantly reversed the reduction in macrophage chemotaxis toward hypoxic tumor supernatant caused by SET deletion (Fig. 6i). Collectively, our data suggest that SET deletion in macrophages releases the activity of PP2A, which subsequently inhibits the activation of ERK and P38 signaling induced by hypoxic tumor supernatant, resulting in the blockade of macrophage migration toward hypoxic tumor supernatant.

### Spatial correlation of SET with macrophages in the hypoxic zones of human tumor samples

**Fig. 7:**
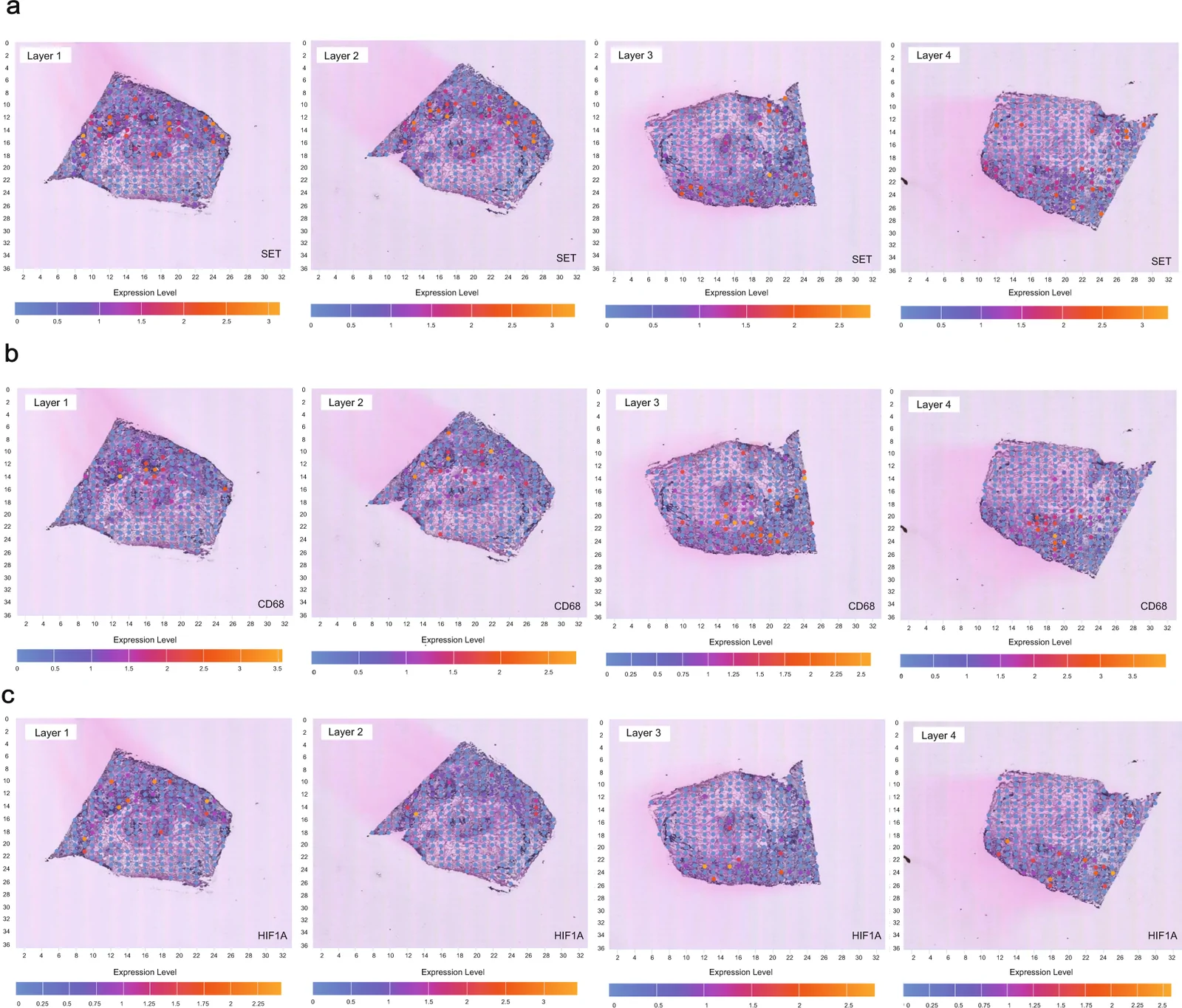
Spatial correlation of SET with macrophages in the hypoxic zones of human tumor samples. **a**–**c** Spatial transcriptome data of breast cancer patients derived from SpatialDB showing the spatial relationships of SET (**a**), CD68 (**b**), and HIF1A (**c**) gene expression. (The dots represent the spatial location of the gene, the color of the dots represents the relative level of gene expression, and each dot is 100 µm in size. Each layer represents a cross-section of the tumor tissue.

To reveal the clinical importance of our findings, we investigated whether SET was spatially related to the distribution of macrophages in hypoxic regions in tumor samples from cancer patients. We analyzed the spatial correlation of SET with the macrophage marker CD68 and the hypoxic zone marker hypoxia-inducible factor 1 subunit alpha (HIF1α) using published spatial transcriptome data from breast cancer patients (Fig. 7a–c). Consistent with our findings, CD68 is mainly distributed in hypoxic regions. Surprisingly, SET was not only significantly overexpressed in hypoxic regions but also colocalized with the high expression of CD68. These data suggest that SET colocalizes with macrophages within human hypoxic tumor regions.

## Discussion

**Fig. 8:**
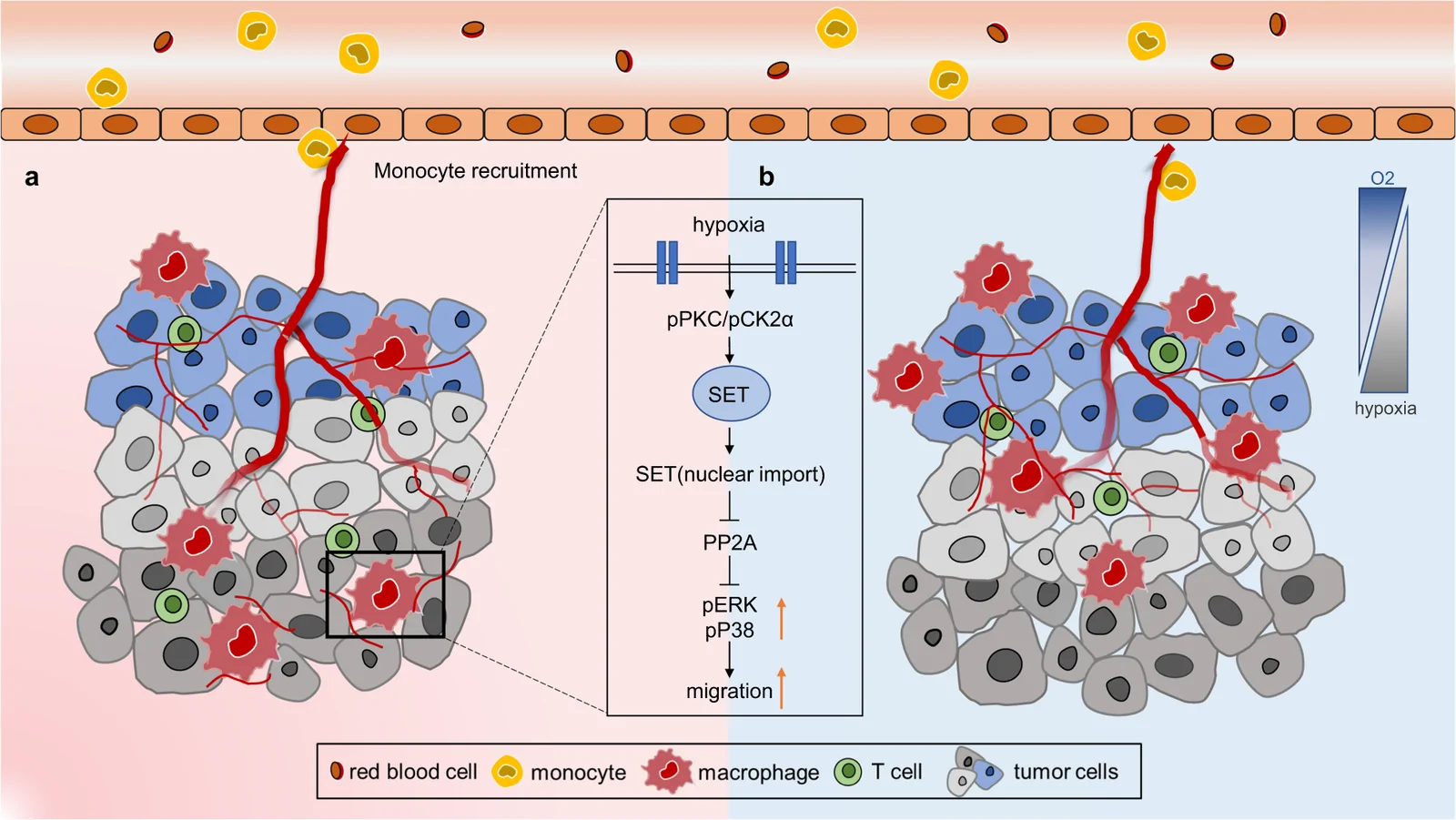
Illustrative working model of SET-mediated macrophage positioning in hypoxic tumor areas. Various chemotactic signals derived from hypoxic tumor regions induced nuclear export of SET by activating PKC-CK2α in macrophages. Cytoplasmic SET accumulation further enhances ERK and P38 signaling by inhibiting PP2A, ultimately driving macrophages to the hypoxic region (**a**). The loss of SET causes TAMs to be retained in the area with blood vessels and away from hypoxic areas, thereby regaining the antitumor phenotype (**b**).

In this study, we demonstrated that the loss of SET in myeloid cells significantly enhanced tumor immunity by impairing intratumoral positioning of TAMs in the hypoxic tumor region, promoting macrophage polarization toward the M1 phenotype, and fostering T-cell infiltration and activation, which reduced tumor burden. Mechanistically, in response to hypoxic tumor supernatant stimulation, SET in TAMs translocated into the cytoplasm via the PKC-CK2α signaling axis. Cytoplasmic retention of SET increased ERK and P38 signaling by inhibiting PP2A and promoted macrophage infiltration into hypoxic areas (Fig. 8). Therefore, our study reveals the key role of SET in controlling the intratumoral localization of TAMs and highlights the clinical importance of SET as a target for cancer immunotherapy.

Due to the complex microenvironment, macrophages within different regions exhibit different phenotypes and functions. The hypoxic zone within the tumor is characterized by a lack of oxygen and nutrients. Macrophages are recruited to the hypoxic zone and undergo metabolic changes to adapt to the microenvironment. Additionally, apoptotic tumor cells in the hypoxic zone release a large number of lipid membrane components, which can be used as energy materials for macrophages. TAMs have been reported to absorb fatty acids through CD36, resulting in the accumulation of intracellular fatty acids and alteration into an immunosuppressive phenotype^42^. Proinflammatory M1-like TAMs use glycolysis to support their functions. Conversely, anti-inflammatory M2-like TAMs use the tricarboxylic acid (TCA) cycle and FAO for their bioenergetic supply^43^. Our study showed that glycolysis-related genes were upregulated and FAO-related genes were downregulated in the TAMs of L/L mice. These metabolism-associated genes were unchanged in SET-depleted BMDMs. Additionally, SET deletion did not cause a significant shift in M1 or M2 polarization in vitro. However, increased M1 TAM polarization in L/L mice was observed. Based on our findings, we hypothesize that macrophage location, metabolic mode, and polarization are functionally correlated and that the location of TAMs may play a central role in regulating the phenotype and function of TAMs.

Hypoxia, which is severe cellular stress, can cause cellular injury and even cell death^44^. A large number of apoptotic and necrotic cells are observed in the hypoxic tumor area^45^. Apoptotic/necrotic cells can release “Find Me” signals that promote macrophage recruitment to the hypoxic region and induce immune tolerance^46^. In our study, we found that the loss of SET impaired the chemotaxis of macrophages in response to apoptotic tumor supernatants (Supplementary Fig. 6b) and reduced the distribution of macrophages in hypoxic regions, which may lead to delayed clearance of apoptotic tumor cells. The impaired clearance of apoptotic tumor cells could activate the immune microenvironment due to the release of intracellular antigens. It has been reported that a specific blockade of the macrophage phagocytic receptor MerTK increases the accumulation of apoptotic tumor cells and triggers a type I interferon response. MerTK blockade increases tumor immunogenicity and potentiates antitumor immunity via the transfer of tumor-derived cGAMP into TAMs through the ATP-gated channel P2X7R and subsequent STING activation^47^. Therefore, we examined whether the loss of SET in macrophages affected their response to necrotic cells. Necrotic tumor cells were prepared and used to stimulate BMDMs. We found that necrotic tumor cells significantly promoted the expression of the proinflammatory factors TNFα, IFNβ, and IL1β in BMDMs from WT and L/L mice (Supplementary Fig. 6c). Therefore, the impaired clearance of apoptotic tumor cells due to the reduction in chemotaxis may also contribute to the antitumor immunity of L/L mice.

Myeloid cells can differentiate into dendritic cells (DCs) and myeloid-derived suppressor cells (MDSCs,) in addition to TAMs in the TME. In the LysM-Cre-LoxP system, SET deletion was present in myeloid cell-derived DCs and MDSCs. We focused on changes in the positioning and function of TAMs. Hypoxic regions also have profound effects on MDSCs and DCs. MDSCs induce T-cell anergy, inhibit the effector phase of CD8^+^ T cells, and promote antigen-specific Treg proliferation, which directly fosters immune tolerance^48^. Recently, Marlene Ballbach et al. demonstrated that hypoxia-mediated upregulation of PD-L1 expression in MDSCs increased MDSC-mediated T-cell tolerance^49^. The effect of hypoxia on the survival, differentiation, activation, and maturation of DCs and their impacts on antitumor immune responses have been well investigated^50^. Indeed, DCs are also diverted by hypoxia from their highly specialized antigen-presenting and T-cell-activating functions. It has been reported that retinoic acid in the TME can predispose monocytes to differentiate into TAMs instead of DCs. Blocking retinoic acid can increase the proportion of DCs, thereby activating tumor immunity^51^. Vinit Kumar et al. showed that depleting TAMs by blocking colony-stimulating factor 1 receptor (CSF1R) could lead to the accumulation of MDSCs in the tumor microenvironment^52^. TAMs, DCs, and MDSCs derived from myeloid cells can be transformed and influenced by each other. Therefore, whether SET deletion in myeloid cells affects the proportion, location, and function of MDSCs and DCs in the TME needs to be further investigated.

CD8^+^ T cells are considered major drivers of antitumor immunity. However, CD8^+^ T cells become functionally exhausted in chronic infections and cancer and are characterized by poor effector function, increased expression of inhibitory receptors, and reduced cytokine production. Hypoxia directly promotes T-cell exhaustion and increases tumor resistance to CTL-mediated lysis. HIF1α enhances the expression of inhibitory receptors, such as PD-1, lymphocyte activating gene 3 (LAG3, also termed CD223), and CTLA-4, in CD8^+^ T cells^53,54^. Additionally, in response to hypoxia, TAMs significantly upregulate the expression of PD-L1 and PD-L2, which bind their receptor PD-1 on T cells and inhibit T-cell effector functions^55^. We found that the expression of PD-L1 and PD-L2 was strongly reduced in TAMs in L/L mice. Consistent with this finding, tumor infiltration and IFNγ secretion by CD8^+^ T cells were significantly increased, facilitating tumor eradication. Moreover, a number of CD8^+^ T cells and macrophages colocalized in hypoxic regions in WT mice. However, both cell populations were decreased in the hypoxic zone in L/L mice. We demonstrated that the loss of SET in TAMs impaired the entry of TAMs into hypoxic tumor regions, but how TAMs affect tumor infiltration and distribution of CD8^+^ T cells remains unclear. Interestingly, Kersten et al. recently showed a similar spatiotemporal connection between TAMs and exhausted CD8^+^ T cells in the inner regions of the tumor^56^. The researchers showed that newly infiltrated antigen-specific CD8^+^ T cells in the TME preferentially localized in TAM-rich areas and were captured during prolonged interactions with TAMs that resulted in the onset of exhaustion programs^57–59^. Due to the important role of TAMs in tumor immunity, we believe that a comprehensive understanding of the crosstalk between TAMs and CD8^+^ T cells would aid to the development of a novel and efficient strategy for cancer immunotherapy.

In our study, we found that the loss of SET inhibited the chemotactic effect of hypoxic tumor supernatant on macrophages. The components of hypoxic tumor supernatant are complex, including various chemokines and released intracellular components. High concentrations of CCL2, CCL5, and CCL8 in hypoxic tumor regions can bind to corresponding receptors on macrophages and induce chemotaxis^60^. Long-term hypoxia can induce apoptosis, and the supernatant of hypoxic tumors may also contain a “Find Me” signal that is released by apoptotic cells to recruit macrophages^61^. In addition, exosomes in hypoxic tumor supernatant may also have chemotactic effects on macrophages. It has been reported that immunomodulatory proteins and chemokines, including CSF-1, CCL2, FTH, FTL, and TGFβ, are highly enriched in exosomes produced by hypoxic tumor cells^62^. These exosomes can affect the recruitment of macrophages and promote M2-like polarization in vitro and in vivo^60^. In addition, hypoxic but not normoxic tumor exosomes enhance oxidative phosphorylation in BMDMs via the transfer of let-7a miRNA, suppressing the insulin-Akt-mTOR signaling pathway^60^. Therefore, it is worth further examining which components in hypoxic tumor supernatant can promote the nucleocytoplasmic shuttling of SET in macrophages to regulate chemotaxis.

## Supplementary information

Supplemental material

Supplemental material

## Data Availability

Data are available upon request from the corresponding author (Changying Guo: guocha@cpu.edu.cn).
