# Author Correction: SET/PP2A signaling regulates macrophage positioning in hypoxic tumor regions by amplifying chemotactic responses

**DOI:** 10.1038/s12276-026-01845-6

**Published:** 2026-09-17

**Authors:** Shaolong Zhang, Jingping Zhou, Pengzhao Shang, Guomeng Zhao, Anlei Wang, Jinlei Mao, Yuhang Tao, Ziyi Chen, Xuehao Wang, Changying Guo

**Affiliations:** 1 School of Life Science and Technology, China Pharmaceutical University, Nanjing, PR China; 2 Hepatobiliary/Liver Transplantation Center, The First Affiliated Hospital of Nanjing Medical University, Nanjing, PR China

Correction to: *Experimental & Molecular Medicine* https://doi.org/10.1038/s12276-022-00867-0, published online 12 October 2022

In this article, the wrong figure appeared as Fig. 5; the figure should have appeared as shown below.

**Figure:**
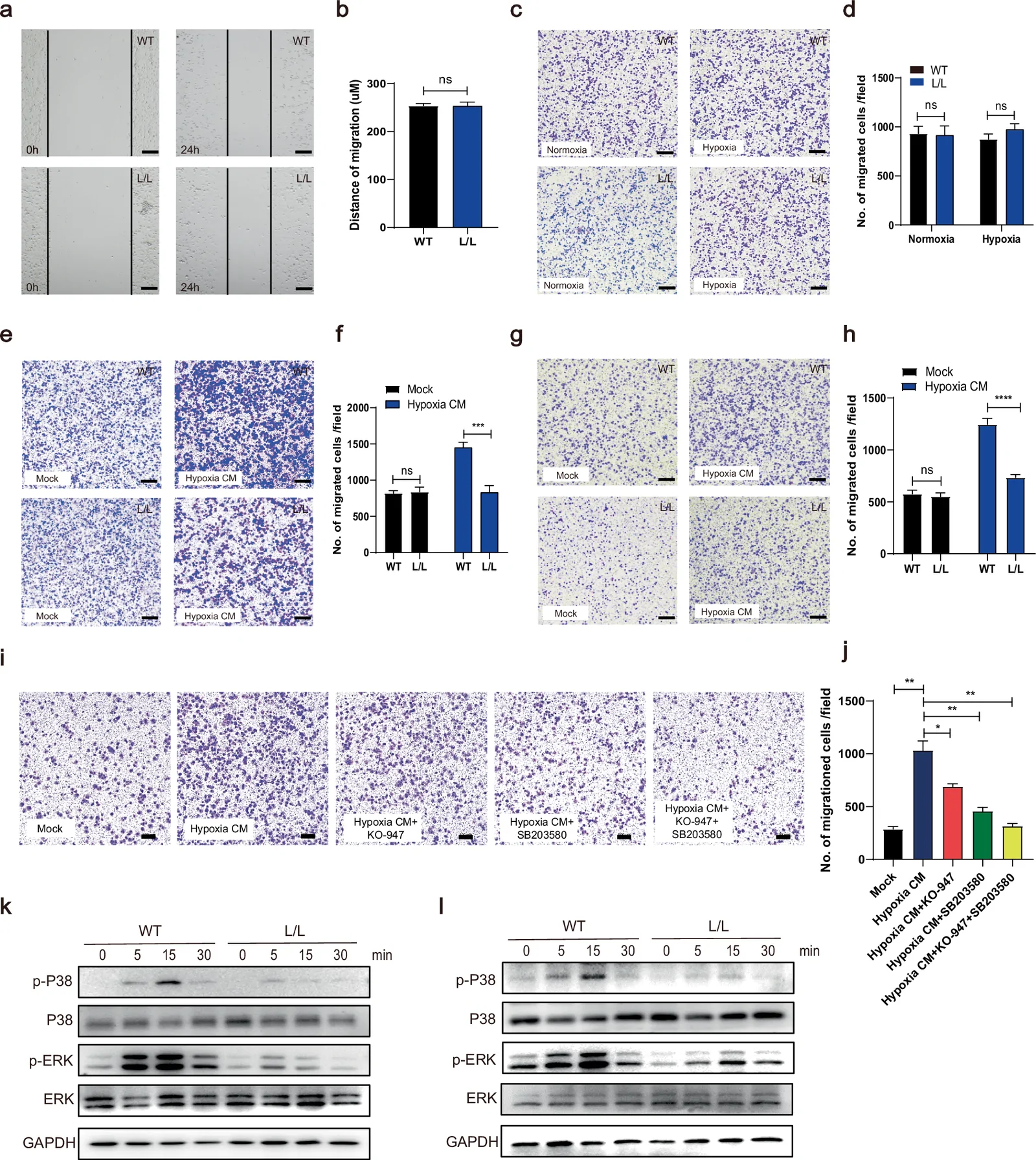

The authors apologize for any inconvenience caused.

The original article has been corrected.

