# Supplementary material for: SET/PP2A signaling regulates macrophage positioning in hypoxic tumor regions by amplifying chemotactic responses
Source: Exp Mol Med. 2022 Oct 12;54(10):1741–55. doi: 10.1038/s12276-022-00867-0 (PMC9636225; doi:10.1038/s12276-022-00867-0)
Supplement: Supplementary file 1 — Supplemental material [file 12276_2022_867_MOESM1_ESM.pdf]

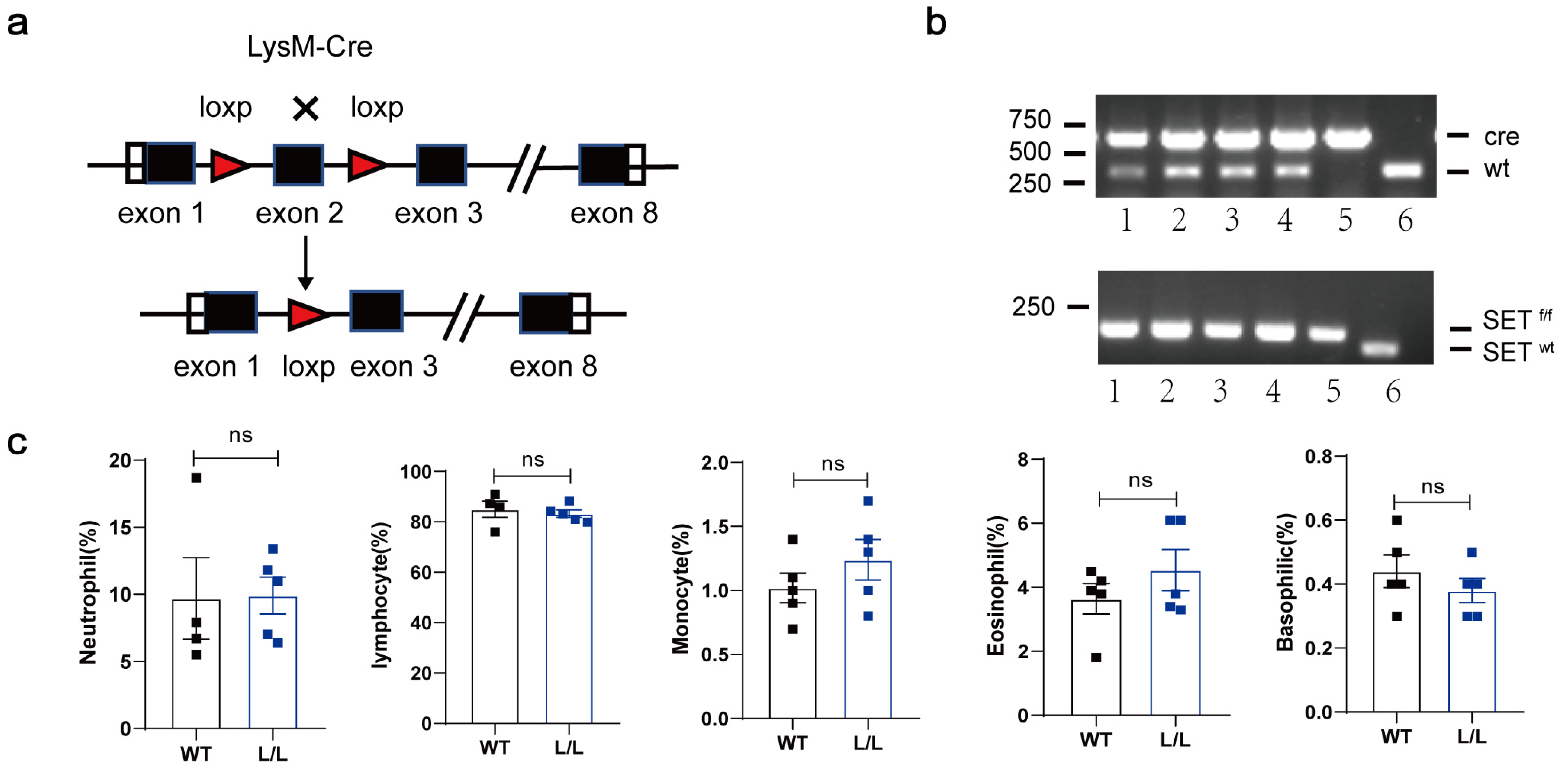

Supplementary Fig. 1. Loss of SET does not affect the proportion of immune cells in the blood

(a) A schematic diagram showing procedures of ablation of SET in myeloid cells.

(b) Genotyping identification of heterozygotes and homozygotes of conditional knockout mice using PCR. Lane 1, 2, 3, 4: LysM-Cre$^{+/-}$,SET$^{fl/fl}$; Lane 5: LysM-Cre$^{+/+}$, SET$^{fl/fl}$; Lane 6: LysM-Cre$^{-/-}$,SET$^{fl/fl}$. LysM gene locus with Cre gene insertion shows about 700 bp band otherwise about 350 bp band (top panel). SET deletion in mice generates a bigger band compared to wild type (bottom panel).

(c) SET deletion does not affect other immune cell development detected by routine blood test of WT and L/L mice.

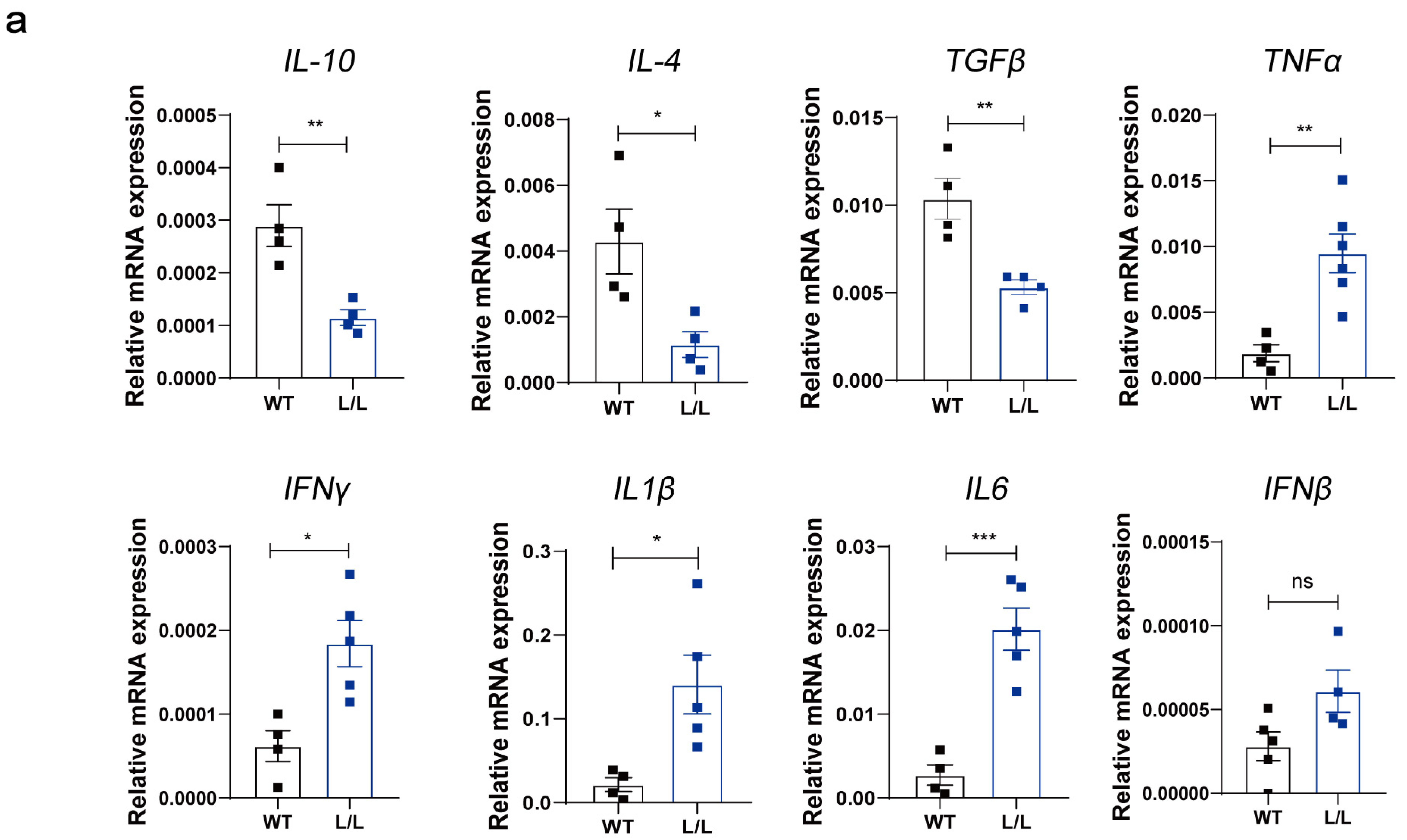

Supplementary Fig. 2. Loss of SET in myeloid cells promotes immune activation of B16F10 tumor

(a) The mRNA levels of the selected cytokine genes, such as IL10, IL4, TGFβ, TNFα, IFNγ, IL1β, IL6 and IFNβ in B16F10 tumor tissues of WT and L/L mice detected by RT-PCR. RT-PCR data show mean ± SEM of at least three biological repeats. Student's *t* test. * $p< 0.05$, ** $p< 0.01$, *** $p< 0.001$, **** $p < 0.0001$.

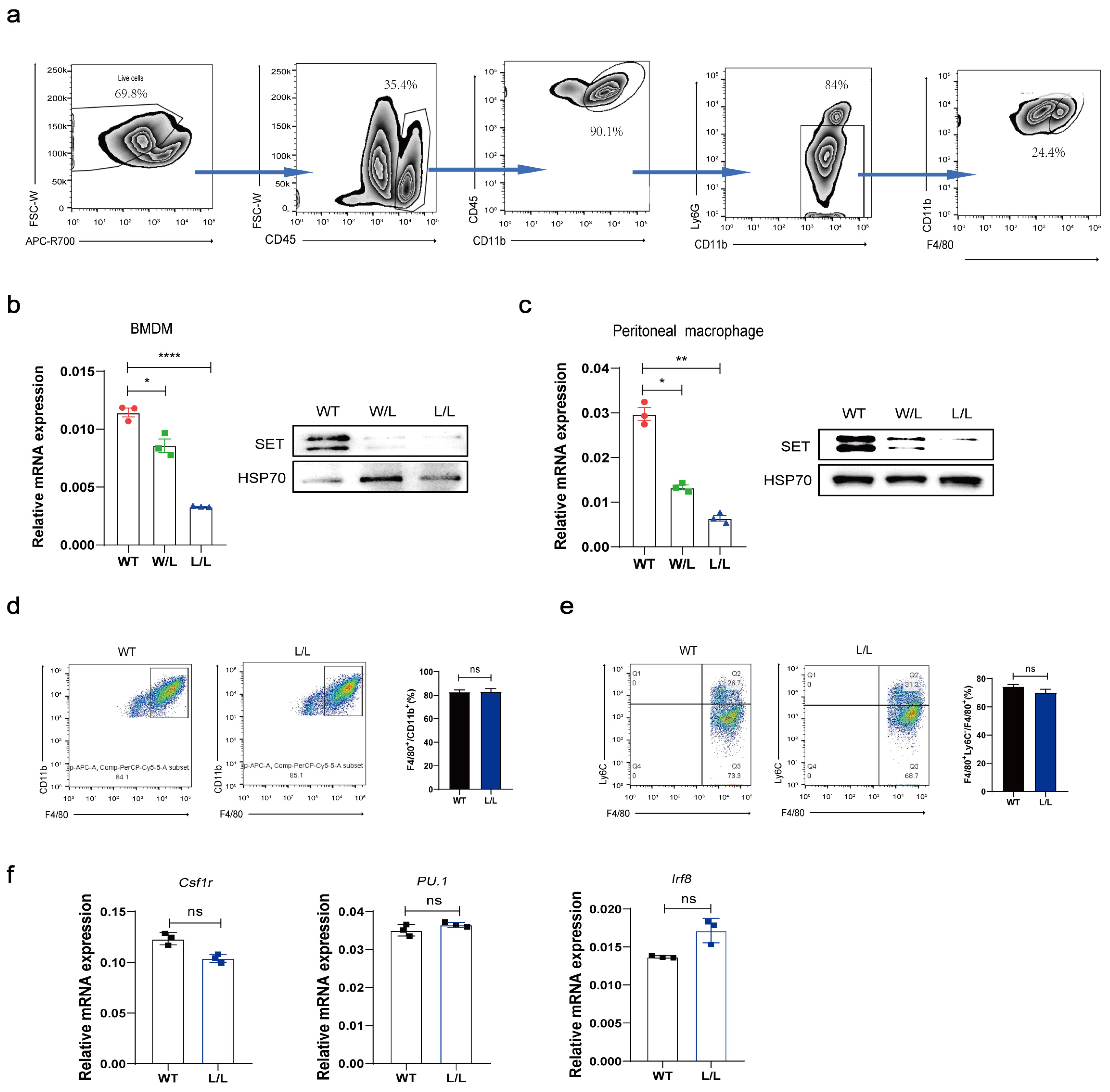

Supplementary Fig. 3. Loss of SET does not affect maturation of macrophages

(a) Flow cytometry defined TAMs populations as CD45$^+$CD11b$^+$LY6G$^-$F4/80$^+$.

(b,c) Deletion of SET in BMDMs and peritoneal macrophages were identified by RT-PCR and western blot assay.

(d,e) SET deletion does not affect BMDMs formation. Bone marrow cells extracted from WT and L/L mice were induced by adding M-CSF (20 ng/ mL) for 7 days. Adherent cells were harvested and determined flow cytometry. F4/80$^+$ cells represent total macrophages (d) and F4/80$^+$LY6C$^-$ represent mature macrophages (e).

(f)The mRNA levels of macrophage maturation-related genes, such as Csf1r, PU.1, and Irf8 detected by RT-PCR. RT-PCR data show mean ± SD of at least three biological repeats. Student's *t* test. * $p< 0.05$, ** $p< 0.01$, *** $p< 0.001$, **** $p < 0.0001$.

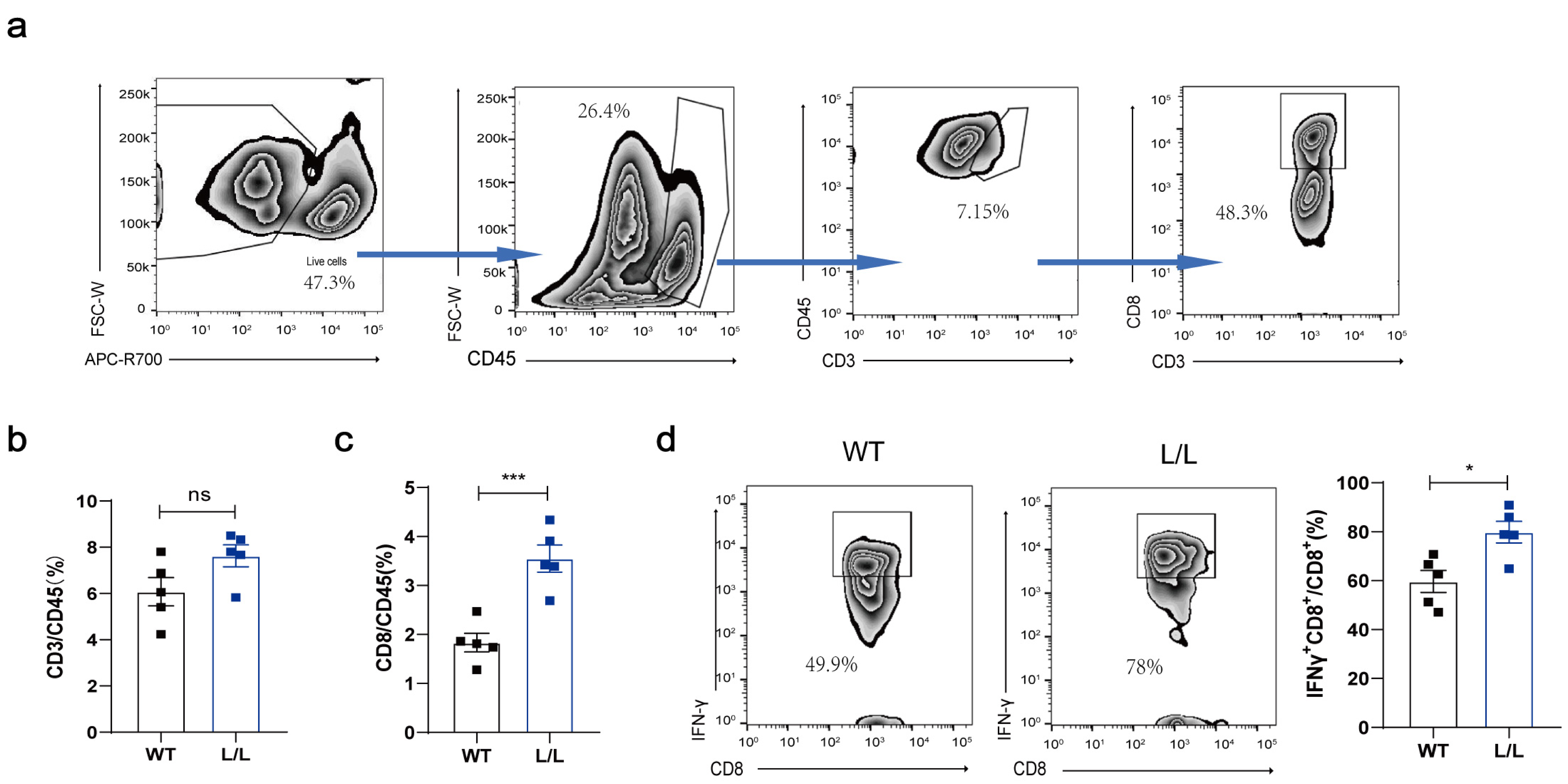

Supplementary Fig. 4. Loss of SET increased infiltration and activation of $CD8^+$ T cells in B16F10 tumor model
(a) Flow cytometry defined CD8+T cells populations as $CD45^+CD3^+CD8^+$.
(b,c,d) FACS analysis of proportion of T cell subtypes CD3+ (b), CD8+ (c) and IFNγ+CD8+ cells (d) in B16F10 tumors.
data show mean ± SEM of at least three biological repeats. Student's *t* test. * $p< 0.05$, ** $p< 0.01$, *** $p< 0.001$, **** $p < 0.0001$.

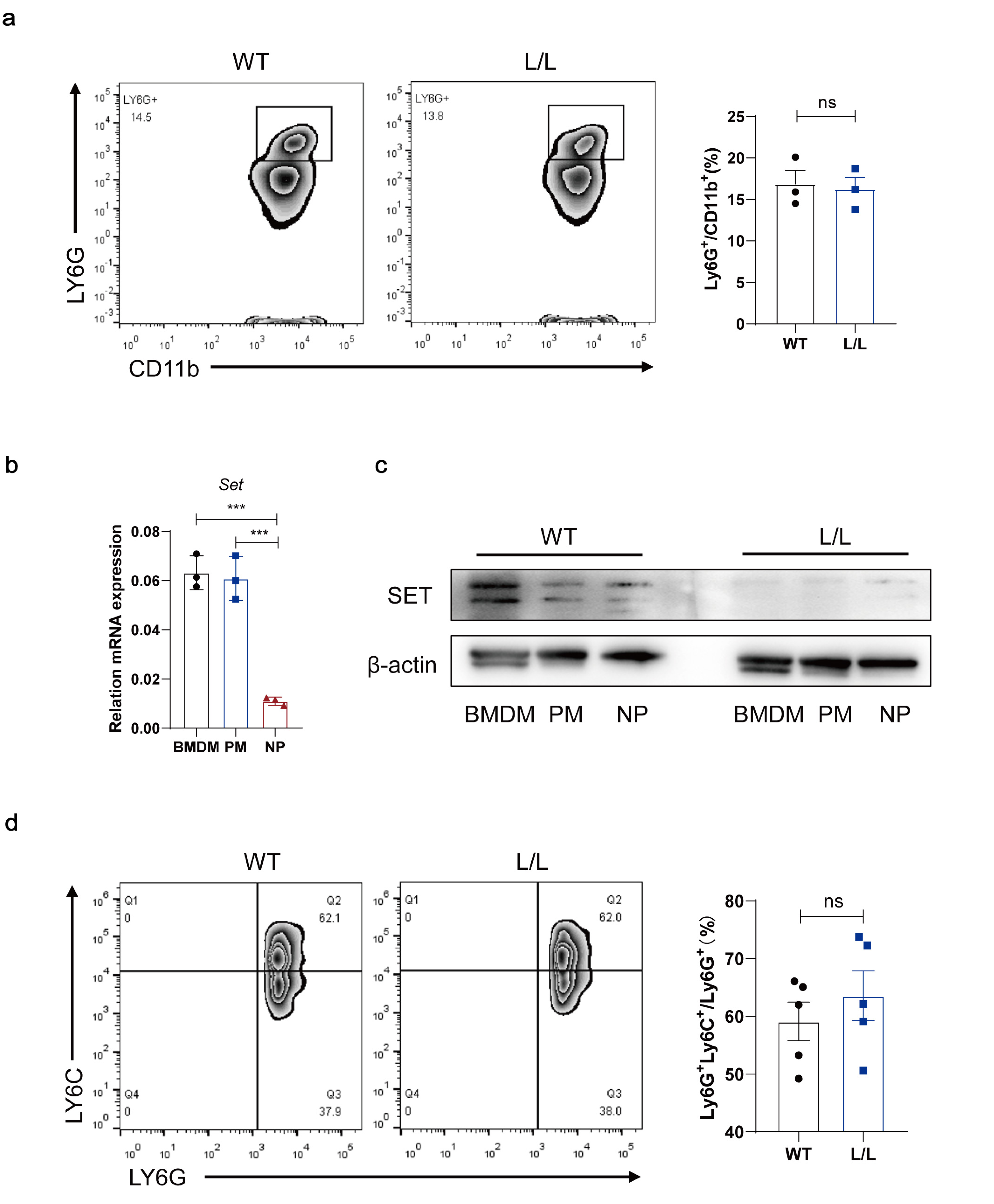

Supplementary Fig. 5. Loss of SET in neutrophils barely affect their plasticity in the TME

(a) FACS analysis of proportion of neutrophil subtypes ($CD11b^+$ $Ly6G^+$) of WT and L/L mice in LLC tumors, respectively.

(b) The mRNA levels of set gene expreesion in bone marrow derived macrophages(BMDMs), peritoneal macrophages(PMs), neutrophils(NPs). RT-PCR data show mean ± SEM of at least three biological repeats. Student's *t* test. * $p< 0.05$, ** $p< 0.01$, *** $p< 0.001$, **** $p< 0.0001$.

(c) Western blot assay showing the SET expreesion in BMDMs, PMs, NPs from WT and L/L mice, respectively.

(d) FACS analysis of N1 subtypes ratio of neutrophils in TME of WT and L/L mice (N1:$Ly6C^{high}Ly6G^+$, N2:$Ly6C^{low}Ly6G^+$) in LLC tumors, respectively.

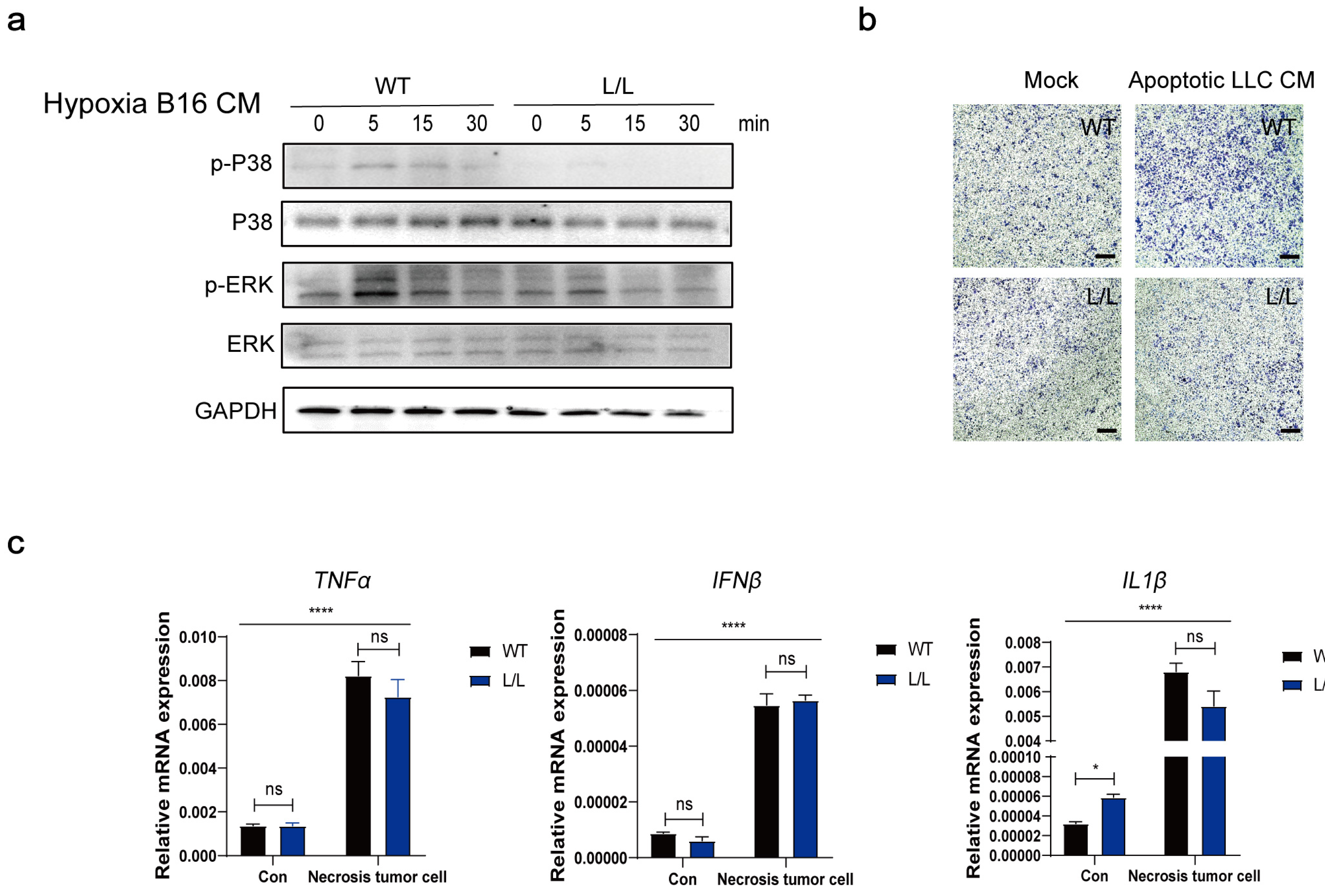

Supplementary Fig. 6. Loss of SET reduces the chemotaxis of macrophages to the supernatant of apoptotic tumor cells

(a)Western blot assay showing the effect of hypoxic B16F10 tumor supernatant on activation of ERK and p38 in BMDMs from WT and L/L mice.

(b)Representative images of transwell migration assays of BMDMs from WT and L/L mice toward the supernatant of apoptotic LLC tumor supernatant. The cells were allowed to migrate for 2 h at 37 °C before staining with crystal violet. Scale bar, 100 μm.

(c)The mRNA levels of TNFα, IFNβ, and IL1β in BMDMs derived from WT and L/L mice stimulated with the supernatant of necrosis tumor cells for 24 h detected by RT-PCR. RT-PCR data show mean ± SEM of at least three biological repeats.

Student's *t* test. * $p< 0.05$, ** $p< 0.01$, *** $p< 0.001$, **** $p< 0.0001$.

Supplementary Table1 Primers used in this study

Name	Forward Primer sequence	Reverse Primer sequence
RT-PCR		
GAPDH	AATGGATTTGGACGCATTGGT	TTTGCACTGGTACGTGTTGAT
TNFα	CAGGCGGTGCCTATGTCTC	CGATCACCCCGAAGTTCAGTAG
IFNγ	ACAGCAAGGCGAAAAAGGATG	TGGTGGACCACTCGGATGA
IL12	ATGGAGTCATAGGCTCTGGAAA	CCGGAGTAATTTGGTGCTTCAC
iNOS	CCCAACGTCATTTCTGTCCGT	TCTACCAGGGGCCGATCATT
IFNβ	AGCTCCAAGAAAGGACGAACA	GCCCTGTAGGTGAGGTTGAT
IL1β	GAAATGCCACCTTTTGACAGTG	TGGATGCTCTCATCAGGACAG
IL6	CTGCAAGAGACTTCCATCCAG	AGTGGTATAGACAGGTCTGTTGG
IL10	GCTGGACAACATACTGCTAACC	ATTTCCGATAAGGCTTGGCAA
IL4	GGTCTCAACCCCCAGCTAGT	GCCGATGATCTCTCTCAAGTGAT
TGFβ	CCACCTGCAAGACCATCGAC	CTGGCGAGCCTTAGTTTGGAC
Arg1	CTCCAAGCCAAAGTCCTTAGAG	GGAGCTGTCATTAGGGACATCA
Fizz	CCAATCCAGCTAACTATCCCTCC	ACCCAGTAGCAGTCATCCCA
Ym1	CAGGTCTGGCAATTCTTCTGAA	GTCTTGCTCATGTGTGTAAGTGA
CD206	CTCTGTTCAGCTATTGGACGC	TGGCACTCCCAAACATAATTTGA
CXCL9	CCGAGGCACGATCCACTACA	CGAGTCCGGATCTAGGCAGGT
CXCL10	CTGAGTGGGACTCAAGGGAT	GTGGCAATGATCTCAACACG
PD-L1	GCTCCAAAGGACTTGTACGTG	TGATCTGAAGGGCAGCATTTC
PD-L2	CTGCCGATACTGAACCTGAGC	GCGGTCAAAATCGCACTCC
Csf1r	TGTCATCGAGCCTAGTGGC	GGTCCAAGGTCCAGTAGGG
PU.1	ATGTTACAGGCGTGCAAAATGG	TGATCGCTATGGCTTTCTCCA
IRF8	AGACCATGTTCCGTATCCCCT	CACAGCGTAACCTCGTCTTCC
Glut1	TCTCGGCTTAGGGCATGGAT	TCTATGACGCCGTGATAGCAG
HK	GGGCATGAAGGGCGTGTCCC	TCTTCACCCTCGCAGCCGGA
PDK1	GGACTTCGGGTCAGTGAATGC	TCCTGAGAAGATTGTCGGGGA
LDH	GGACAGTGCCTACGAGGTGAT	GGATGCACCCGCCTAAGG
PGK1	GGAAGCGGGTCGTGATGA	GCCTTGATCCTTTGGTTGTTTG
CPT1A	TCCAGTTGGCTTATCGTGGTG	TCCAGAGTCCGATTGATTTTTGC
EHHDH	ATGGCTGAGTATCTGAGGCTG	GGTCCAAACTAGCTTTCTGGAG
HADH	TCAAGCATGTGACCGTCATCG	TGGATTTTGCCAGGATGTCTTC
ECHS1	AGCCTGTAGCTCACTGTTGTC	ATGTACTGAAAGTTAGCACCCG
CD36	AGATGACGTGGCAAAGAACAG	CCTTGGCTAGATAACGAACTCTG

Supplementary Table2 Antibodies used in this study

Name	Purpose	resource	Catlog. #
Fixable viability stain 700	Flow cytometry	BD Pharmingen	564997
CD16/32-BV510	Flow cytometry	Biolegend	101333
CD3e-percp cy5.5	Flow cytometry	BD Pharmingen	551163
CD4-PE	Flow cytometry	Biolegend	100407
CD8-APC-CY7	Flow cytometry	BD Pharmingen	561967
IFN γ-APC	Flow cytometry	BD Pharmingen	505810
CD45-FITC	Flow cytometry	Biolegend	103108
CD11b-Percp-cy5.5	Flow cytometry	Biolegend	101227
F4/80-APC	Flow cytometry	Biolegend	123116
LY6G-APC-CY7	Flow cytometry	Biolegend	127623
LY6C-PE	Flow cytometry	Biolegend	128007
CD206-BV421	Flow cytometry	Biolegend	141717
SET	WB	Abclonal	A12502
P-P38	WB	CST	4511
P38	WB	CST	8690
ERK	WB	CST	4695
P-ERK	WB	CST	4370
GAPDH	WB	Proteintech	60004-1-Ig
Lamin B	WB	Proteintech	12987-1-AP
HSP70	WB	Proteintech	10995-1-AP
P-PKC	WB	Abclonal	AP0191
P-CK2α	WB	Arigo	ARG51843
CK2α	WB	Proteintech	10992-1-AP
